# The uptake of selenium by perennial ryegrass in soils of different organic matter contents receiving sheep excreta

**DOI:** 10.1007/s11104-023-05898-8

**Published:** 2023-02-02

**Authors:** Pei-Tzu Kao, Heather L. Buss, Steve P. McGrath, Tegan Darch, Helen E. Warren, Michael R. F. Lee

**Affiliations:** 1 Rothamsted Research, North Wyke, Okehampton, EX20 2SB Devon UK; 2 School of Earth Sciences, University of Bristol, Bristol, BS8 1RJ UK; 3 Rothamsted Research, Harpenden, AL5 2JQ Hertfordshire UK; 4 Alltech Bioscience Centre, Sarney, Summerhill Road, Dunboyne, Co. Meath Ireland; 5 Harper Adams University, Newport, TF10 8NB Shropshire UK

**Keywords:** Fertilizers, Manure, Microbial reduction, Grassland soil, Pasture, Ruminants

## Abstract

**Background and aims:**

The intake of selenium, an essential element for animals and humans, in ruminants is largely determined by selenium concentration in ingested forages, which take up selenium mainly from soil. Ruminant excreta is a common source of organic fertilizer, which provides both nutrients and organic matter. This study aims to unentangle the unclear effect of applying different types of ruminant excreta in soils of different organic matter contents on selenium uptake by forage.

**Methods:**

Perennial ryegrass (*Lolium perenne*) was grown in soils of different organic matter contents. Urine and/or feces collected from sheep fed with organic or inorganic mineral supplements, including selenium, were applied to the soils. The selenium in the collected samples were analyzed using ICP-MS. The associated biogeochemical reactions were scrutinized by wet chemistry.

**Results:**

The application of urine and/or feces resulted in either the same or lower selenium concentrations in perennial ryegrass. The excreta type did not affect total selenium accumulation in grass grown in low organic matter soil, whereas in high organic matter soil, feces resulted in significantly lower total selenium accumulation than urine, which was attributed to a possible interaction of selenium sorption in soil and microbial reduction of Se.

**Conclusion:**

This one-time excreta application did not increase, but further decrease in some treatments, selenium concentration and accumulation in the perennial ryegrass. Consequently, to increase ruminant selenium intake, supplementing selenium directly to animals is more recommended than applying animal manure to soil, which might drive selenium reduction and decrease selenium uptake by grass.

**Supplementary Information:**

The online version contains supplementary material available at 10.1007/s11104-023-05898-8.

## Introduction

Selenium (Se) is essential for all animals as a component of selenocysteine (Se-Cys), which is present in vital enzyme systems such as glutathione peroxidase, which functions as an antioxidant protecting cell membranes (Eswayah et al. 2016; Tórtora-Pérez 2010). Therefore, deficiency of Se can result in a range of functional disorders such as white muscle disease, poor immune system function and even infertility due to oxidative damage of cellular and mitochondrial membranes (Lee et al. 2018; Tórtora-Pérez 2010). Animals must acquire Se from the diet, and for ruminant livestock this relates predominately to the intake of forage. However, the average concentration of Se in UK pastures is significantly lower than the dietary requirement of ruminants, resulting in a need to supplement (Kao et al. 2020). Unfortunately, intake of Se between deficiency and toxicity (chronic) is typically narrow for most animals, including ruminant livestock (< 0.1 – 5.0 mg kg^−1^ dry matter; Lee et al. 2019a).

Se toxicity and deficiency typically occur in areas with seleniferous soils and soils with particularly low Se contents, respectively (Winkel et al. 2015). However, total soil Se concentration are not always positively correlated with soil Se bioavailability. For example, both Kaschin-Beck disease and Keshan disease, two main human diseases related to Se deficiency, have been reported in China where the soil Se content ranges from < 0.1 mg kg^−1^ (extremely low-Se) to 0.3 mg kg^−1^ (Se-adequate) (Wang and Gao 2001). The Se concentration in plants in China was reported to be significantly dependent on the soil soluble Se instead of total soil Se (Fernández-Martínez and Charlet 2009; Wang and Gao 2001). The soil soluble Se is generally governed by the sorption of iron (Fe) or aluminum (Al) oxides and/or soil OM, which is affected by soil pH (Fernández-Martínez and Charlet 2009). For example, the high Se accumulation in the top soil in northeastern China is likely related to the high soil OM content, whereas the high Se in soils (> 0.4 mg Se/kg) of the Yangtze River and Zhujiang River drainage areas in China is likely related to the acidic and reductive soil conditions, in which Se tends to occur in the form of selenite (SeO_3_^2−^) and accumulates with Fe and Al as insoluble oxides such as Fe_2_(SeO_3_)_3_ and Fe_2_(OH)_4_SeO_3_ (Wang and Gao 2001). In the soils sampled from 48 fields of different soil properties in Finland, it was reported that the fractionations of soil Se from the different fields followed a similar pattern and most of the soil Se (ca. 40%) was in the organically associated fraction (Keskinen et al. 2011).

Speciation also influences flux of Se in the environment and its availability to organisms (Fernández-Martínez and Charlet 2009). SeO_3_^2−^ and selenate (SeO_4_^2−^) and seleno-amino acids, e.g., selenomethionine (Se-Met) and Se-Cys are plant-available (Kikkert and Berkelaar 2013), whereas the elemental Se or selenides formed with Se(-II) have low solubilities (Séby et al. 2001). Sulfate (SO_4_^2−^) and phosphate (PO_4_^3−^) are both known to compete with SeO_4_^2−^ and SeO_3_^2−^, respectively, for the uptake sites on plant roots (Ávila et al. 2020; Hopper and Parker 1999). The uptake of SeO_4_^2−^ by plants from soils is typically greater than SeO_3_^2−^, which is thought to relate to the different absorption mechanisms in plant roots, with the former predominately transported actively through sulfate transporters and the latter predominately passively absorbed (Fernández-Martínez and Charlet 2009; Sors et al. 2005). However, it has been proposed that SeO_3_^2−^ can be transported actively through cell membranes, evidenced by a similar response to CCCP (carbonyl cyanide 3-chlorophenylhydrazone), a metabolic inhibitor that interferes with the proton gradient and depolarizes the plasma membrane (Li et al. 2008). Therefore, it was argued that dissolved SeO_3_^2−^ is at least as available as SeO_4_^2−^ for uptake by wheat, but because SeO_3_^2−^ is usually adsorbed more strongly by the soil solid phase (e.g. Fe oxides/hydroxides) than SeO_4_^2−^, it is less abundant than SeO_4_^2−^ in soil solutions (Li et al. 2008). Seleno-amino acids (Se-Met and Se-Cys) are more efficiently taken up by plants than inorganic Se because they can be actively taken up through amino-acid transporters (Kikkert and Berkelaar 2013). Se speciation mobility and bioavailability in the environment are substantially affected by microorganisms, which can transform Se species via reduction, methylation, oxidation, and demethylation (Eswayah et al. 2016).

Continuous use of inorganic chemical fertilizers may lead to soils deficient in micronutrients such as Se, with animal manure seen as an OM-rich alternative to overcome such deficiencies (Power and Prasad 1997). However, it is not clear how bioavailable manure-derived micronutrients are within pasture soils. To remediate Se-contaminated soils, amendment with manure or straw was reported to decrease Se accumulation in wheat (*Triticum aestivum*) grown in seleniferous soils and to enhance the removal of Se from the soils via Se volatilization (Li et al. 2017). However, Øgaard et al. (2006) reported an increase in Se accumulation in wheat when applying cattle slurry to a peat soil with typical levels of Se (0.23–0.28 mg Se kg^−1^ soil; pH = 6.8), but no difference was observed in a loam soil (0.26 mg Se kg^−1^ soil; pH = 6.0). Smažíková et al. (2019) reported that the adsorption coefficient (*Kd*) values of soils remained constant after the application of vermicompost and digestate in fluvisol, chernozem and luvisol soils, implying that Se mobility in the soils were not significantly altered by adding the organic material. These variable responses to applying organic materials to soil on Se accumulation in wheat can be explained by different effects of dissolved organic carbon (DOC) and soil OM. The release of DOC can increase the mobility of Se by competing for sorption sites whereas the input of soil OM can decrease Se bioavailability via immobilization effects (Li et al. 2017). The results of Øgaard et al. (2006) imply that the effect of applying organic materials to soils on Se accumulation in plants is also associated with the pH and other soil properties. However, such interactions between soil properties and Se from organic materials applied to soils, and resulting effects on Se uptake by plants, still need further investigation.

In grazing pasture systems, soils receive nutrients and OM from animal manures directly. According to the 2017 data from FAO (Data source: http://fao.org/faostat, data selection: Data/Emissions-Agriculture/ Manure left on Pasture, options: regions (World + [Total])/elements (Manure[N content])/ Items (All animals > [List])/Years (2017)), more than 80% of the N input to pastures came from the manures of ruminants, reflecting the regular on-farm application of ruminant manures (Kao et al. 2020). Recently, we showed that 51 to 64% of total Se consumed by sheep was excreted either through urine or feces (unpublished data). Urine and feces can therefore be an important source of available Se. However, urine and feces are significantly different in their physiochemical properties, such as nutrient content, nutrient release rate (Blair et al. 1994) and pH (Morgante et al. 2009). Therefore, we found it necessary to separate the urine from the feces in order to investigate the potential impact of their different physicochemical properties and potentially different Se forms on the ultimate Se uptake by plants after they are applied to soils. The impact of different forms (organic versus inorganic) of mineral supplements in animal feed on the excretion of Se in urine and feces was previously studied (unpublished data). The form of the mineral supplements (specified in Table 1) supplied to sheep did not significantly impact the partitioning of Se between urine and feces, but it influenced the partitioning of Se in some sequentially extracted fractions of feces. The potential impact of different forms of supplemental minerals on Se uptake by perennial ryegrass through excreta was therefore also further investigated in the present study.

**Table 1:** The sources and the physiochemical properties of the applied urine and feces

Treatment symbols*		U-I	F-I	U–O	F-O
Form of mineral supplement given to the sheep		Inorganic minerals (sodium selenite, copper sulfate pentahydrate, zinc oxide and manganese oxide)		Organic minerals (selenized yeast (Selplex®), copper, zinc, and manganese chelate of protein hydrolysate (Bioplex®))	
Excreta type		Urine	Feces	Urine	Feces
Dry matter		18.3 ± 0.29 g L^−1^	22.2 ± 2.07 g kg^−1^	13.8 ± 0.066 g L^−1^	25.9 ± 7.43 g kg^−1^
pH (Measurement method)		9.40	8.10	9.49	8.10
		(Direct measurement)	(5 g sample in 20 mL water)	(Direct measurement)	(5 g sample in 20 mL water)
Total element concentrations	Nitrogen	7.04 ± 1.058 g L^−1^	Not measured	7.36 ± 1.812 g L^−1^	Not measured
	Phosphorus	3.50 ± 0.860 mg L^−1^	12.3 ± 0.78 g kg^−1^	3.64 ± 0.954 mg L^−1^	12.2 ± 0.33 g kg^−1^
	Selenium	29.8 ± 5.46 μg L^−1^	576 ± 23.4 μg kg^−1^	22.2 ± 3.65 μg L^−1^	505 ± 21.2 μg kg^−1^
	Sulphur	1176 ± 129.2 mg L^−1^	3.52 ± 0.077 g kg^−1^	1062 ± 125.2 mg L^−1^	3.70 ± 0.096 g kg^−1^
Se concentrations in fractions of sequential extraction of feces (μg kg^−1^; % in total fractions)	Fraction 1: Water- and acid- soluble		76.3 ± 5.56 (33.0%)		71.0 ± 5.00 (32.6%)
	Fraction 2: Exchangeable		11.2 ± 0.85 (4.7%)		10.8 ± 0.41 (4.8%)
	Fraction 3: Sorption on OM and/or Fe/Al hydroxides		74.4 ± 1.94 (32.3%)		67.3 ± 2.91 (29.1%)
	Fraction 4: Residual		76.8 ± 8.76 (30.0%)		83.1 ± 8.56 (33.5%)

*U  Urine; *F*  Feces; *I* Inorganic; *O* Organic

In the current study, we hypothesized that applying sheep excreta in soils could affect the Se uptake by perennial ryegrass, and the impact was from two main causes: (1) the different chemical forms of Se supplement given to the sheep (2) the interaction between the excreta and soils of different OM status. We also hypothesized that Se accumulation would be lower in the perennial ryegrass grown in the soil of higher OM due to Se adsorption onto OM. To understand the mechanism of the potential impact of soil-excreta interaction, we applied the urine and feces, either separately or combined to test whether the potential interaction could be driven by the changes of (1) soil pH, (2) extractable Se: sulfur (S): phosphorus (P) in the soil, (3) contrasting available Se provided via urine or feces, (4) physiochemical properties of urine and feces that result in different decomposition rates and different mobility of nutrients in the soils after the application.

Samples of urine and/or feces from the sheep, offered either organic or inorganic mineral supplements (including 0.37 mg Se animal^−1^ d^−1^ from Se-yeast or SeO_3_^2−^; aligned with the EU regulation of the maximum permitted allowance of organic Se at 0.2 mg Se kg^−1^complete feed at 12% moisture), were applied to soils of the same soil type but differing in organic carbon contents (15.6 and 35.6 g kg^−1^). Perennial ryegrass (*Lolium perenne* L.) is the most important agricultural grass species in temperate grasslands (Bolaric et al. 2005). Perennial ryegrass (cv. AberMagic) was grown in the soils and harvested 3 times with an interval of 2 w. Selenium uptake, including Se concentration and Se accumulation (concentration in above ground tissue x above ground biomass), in perennial ryegrass was analyzed using ICP-MS following an acid-digestion.

## Materials and methods

### Experiment design

Urine and feces from sheep offered supplemental minerals of different forms (organic or inorganic, Table 1) on a forage: concentrates ration = 60:40 for 2 w, were collected and applied either separately or in combination to experimental soils in pots. The soils were collected from an arable field (Great Harpenden) or a nearby grassland field (Weighbridge Piece) at Rothamsted Research, Harpenden, UK (51.81°N, 0.35°W) to provide the same soil type with low or high soil OM content, respectively. The soils from the two fields were Batcombe Series (Clayden and Hollis 1984) but had significant differences in soil OM content (Table 2). The inorganic carbon contents of the two soils were negligible. The soils of Great Harpenden and of Weighbridge were sampled to 23 cm and 10 cm depth, respectively, to magnify the difference in soil OM content. The properties of each soil are shown in Table 2.

**Table 2:** The soil physiochemical properties and the analytical methods

Soil properties		Great Harpenden (L)	Weighbridge Piece (H)	Analytical method and/or instrument
Sampling depth		to 23 cm depth	to 10 cm depth	-
Soil texture	Sand (2.00–0.063 mm)	22%	19%	pipette sampling method
	Silt (0.063–0.002 mm)	49%	54%	
	Clay (< 0.002 mm)	29%	27%	
	Textural class	Clay loam	Silt clay loam	
Redox- active oxides (mg kg^−1^)	Al	1099 ± 8.9	1087 ± 6.7	Extraction using 0.114 M ammonium oxalate and 0.086 M oxalic acid (Rayment and Lyons 2011; Schwertmann 1964; Sparks et al. 2020)
	Fe	4528 ± 56.6	8200 ± 48.7	
	Mn	1506 ± 25.9	1436 ± 23.0	
	P	360 ± 7.4	1003 ± 8.7	
Bulk density in field (g cm^−3^)		1.4 ± 0.01	1.0 ± 0.06	-
Soil pH		6.38 ± 0.012	6.31 ± 0.016	10 g soil in 25 mL ultra-pure water (18 MΩ)
Organic carbon (g kg^−1^)*		15.6 ± 0.39	35.6 ± 0.24	Elemental analyser (NA-1500, Carlo-Erba®)
Total nitrogen (g kg^−1^)		0.02 ± 0.001	0.03 ± 0.001	
2 M KCl extractible N (mg-N kg^−1^ soil)		8.7 ± 0.75	15.9 ± 2.82	Photometric analyzer (Aquakem 250, Thermo Scientific®)
Total P (g kg^−1^)		0.81 ± 0.013	1.67 ± 0.058	Acid digestion using aqua regia and analysed with ICP-MS or ICP-OES
Total Fe (g kg^−1^)		33.6 ± 0.61	27.3 ± 0.89	
Total Mn (g kg^−1^)		1.79 ± 0.060	1.61 ± 0.058	
Total Cu (mg kg^−1^)		17.7 ± 0.22	24.1 ± 0.93	
Total Zn (mg kg^−1^)		72.1 ± 2.22	101 ± 3.4	
Total Se (μg kg^−1^)		782 ± 14.0	865 ± 26.6	

*A preliminary test showed that the arable soil and the grassland soil only contained ca. 0.01% and 0.02% inorganic carbon, respectively, which were little in the total carbon content in the soils. Therefore, the presented total carbon contents are close to the content of total organic carbon

In total, there were 14 treatment combinations to reflect a multifactorial experiment: two soils sampled from either a grassland (of higher soil OM, H) or an arable land (of lower soil OM, L), applied with feces (F) or urine (U) or urine + feces (UF) which were collected from sheep that were supplemented either organic (O) or inorganic (I) supplemental minerals (in total 12 treatment combinations), plus the soils with no application of urine and feces (G-CK and A-CK) (Table 3). The experiment followed a Randomized Complete Block Design. There were four blocks, and each block included one replicate of the 14 treatments. The inclusion of the blocks was designed to consider the potential covariant from the environment and the difference in the time taken for irrigation and sampling between each block.

**Table 3:** Symbols used for the different treatments

Soil	Excreta type	Forms of supplemental minerals to sheep	Treatment symbols
Soil from arable land (L)	Control check (CK): no excreta applied		L-CK
	Feces (F)	Inorganic (I)	L-F-I
		Organic (O)	L-F-O
	Urine (U)	Inorganic (I)	L-U-I
		Organic (O)	L-U–O
	Urine + feces (UF)	Inorganic (I)	L-UF-I
		Organic (O)	L-UF-O
Soil from grassland (H)	CK: no manure applied		H-CK
	Feces (F)	Inorganic (I)	H-F-I
		Organic (O)	H-F-O
	Urine (U)	Inorganic (I)	H-U-I
		Organic (O)	H-U–O
	Urine + feces (UF)	Inorganic (I)	H-UF-I
		Organic (O)	H-UF-O

Monoculture perennial ryegrass (*Lolium perenne* cv. AberMagic), a grass forage species selected from the recommended list for grazing in England and Wales (AHDB 2020), was used to study Se uptake by forages. The perennial ryegrass was harvested from each pot three times to study the cutting time effect on Se uptake. The experiment was carried out in a temperature-controlled room maintained at 20℃ during the day and at 16℃ at night. An artificial LED light source gave 16 h of light a day with light illuminance = 33,000–57,000 lx (measured from the soil surface using Digital Lux Meter (LX1330B, Dr.meter®)), which mimics the outdoor light under direct sunlight.

### Pot design

**Fig. 1:**
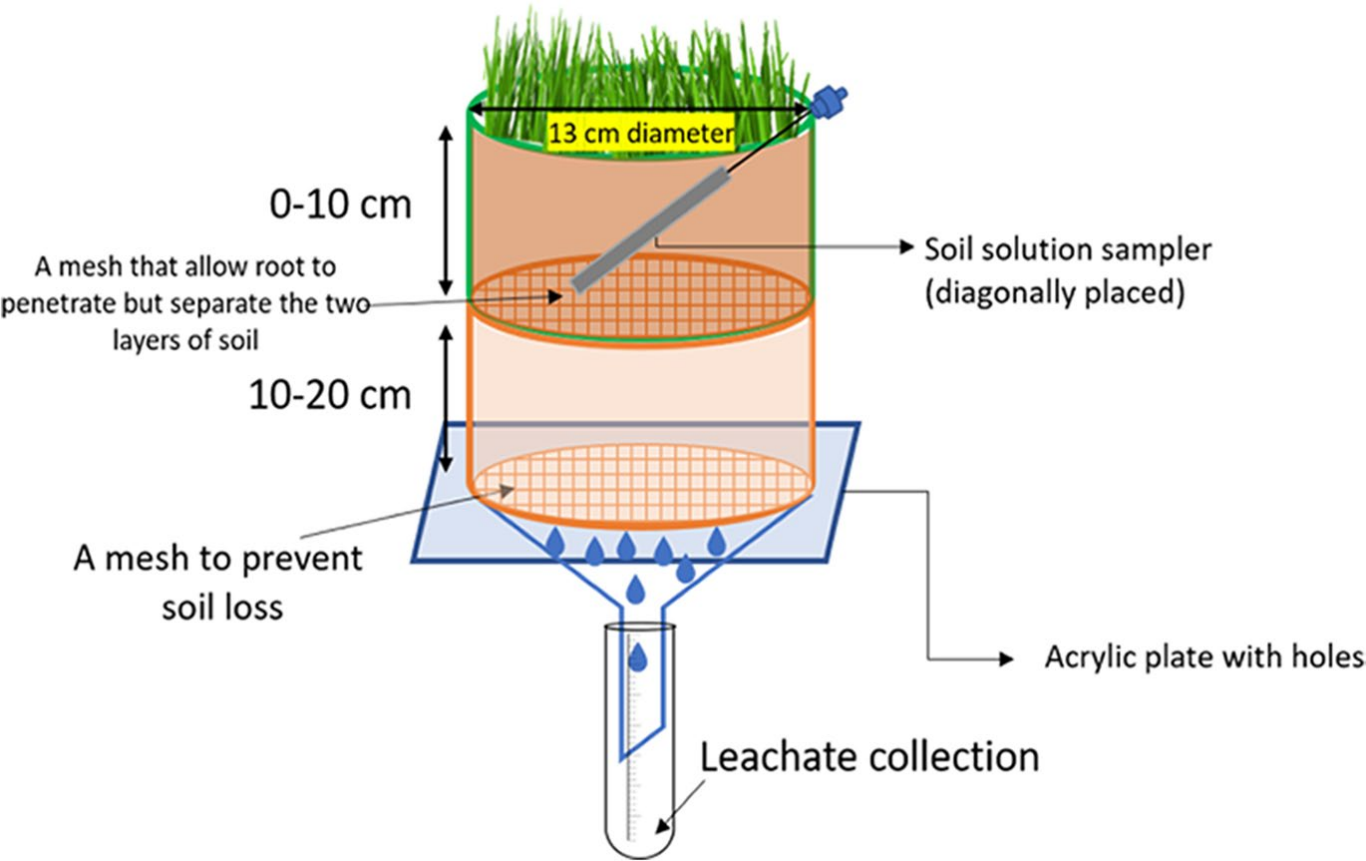
The pot used in this study was composed of a soil column with two layers and a leachate collection apparatus

Each pot (Fig. 1) was made from a cut PVC water pipe, 13 cm inner diameter by 22 cm depth. In the middle of the column was a plastic mesh (pore size = 1.5 mm × 1.5 mm) used to divide the soil column into two layers. The sheep excreta were applied into the top layer. At the bottom of the pot another plastic mesh with the same pore size was set to prevent soil loss but allow leachate to pass through. Underneath the soil column was an acrylic plate with holes to hold the soil but allowing the leachate to filter through to the collecting container below. A Rhizon soil solution sampler (pore size = 0.15 μm, length = 10 cm, diameter = 2.5 mm, with stainless steel strengthening wire and 10 cm PVC-tube; Rhizosphere Research Products®, Netherlands) was placed diagonally in the top-layer of soil to collect soil solution for pH measurement.

### Application densities of urine and feces

The application of urine and feces to the pots was designed to reflect the amounts that soils receive in field. The urine application density (0.52 mL cm^−2^ = 70 mL per pot) was calculated according to the observed areas of urine patches in a field (290 cm^2^) reported by Doak (1952) and the average volume of each urination of sheep (150 mL) reported by Sears et al. (1942). Using the urine application rate and the excretion ratio of urine and feces (4.764 mL g-DM^−1^) from a previous sheep experiment (data unpublished), the feces application density was calculated as 0.11 g-DM cm^−2^ (= 15 g-DM feces per pot). Due to the high moisture content of feces, 100 g moist feces was applied to make sure that the total DM of feces was more than the pre-determined application amount (15 g-DM). The total input of Se from the soil and excreta in each pot is shown in Table 4.

**Table 4:** Total input of soil, feces and urine and the input of Se from the excreta

Treatments	Soil weight (kg-DM pot^−1^)	Feces moisture* content (g moisture/g fresh feces *100%)	Feces input (g-DM pot^−1^)	Urine input (mL pot^−1^)	Se from the excreta (μg)
L-CK	2.68	-	-	-	-
L-F-I		78%	22	-	12.70
L-F-O		74%	26	-	15.75
L-U-I		-	-	70	2.087
L-U–O		-	-	70	1.553
L-UF-I		78%	22	70	14.77
L-UF-O		74%	26	70	17.31
H-CK	2.64	-	-	-	-
H-F-I		78%	22	-	12.70
H-F-O		74%	26	-	15.75
H-U-I		-	-	70	2.087
H-U–O		-	-	70	1.553
H-UF-I		78%	22	70	14.77
H-UF-O		74%	26	70	17.31

*The moisture contents of soil and feces were measured on the day of filling the pot. The values presented were the average of three replicates

### Preparation of the soils, the sheep excreta and the irrigation water

The soils were air-dried in a greenhouse and sieved through a 2 mm stainless-steel mesh. In total, 2.80 kg air-dried soil (bulk density of 0.90 g cm^−3^) was put into each pot. Due to the nature of the study, there were no rain drops and less microfaunal activity in the pots than in the field, which would normally help decompose the applied sheep excreta. Therefore, the feces in this study were crumbled and integrated into the top layer of soil instead of being laid on the soil surface, whereas the urine was applied by pouring evenly on the soil surface on day 0. The fresh feces (DM%; Table 4) was crumbled into smaller pieces by passing the feces through a stainless-steel mesh (hole size = 11 mm). The irrigation water was prepared by adding 1 mL artificial rainwater (ARW) stock and making up to 1 L with Milli-Q water. The formulation of ARW stock (Table S3) was based on the mean values of element contents from monthly rainwater samples collected over a ten-year period at Rothamsted Research’s North Wyke site (Darch et al. 2019).

### Experiment timeline and pot management

**Fig. 2:**
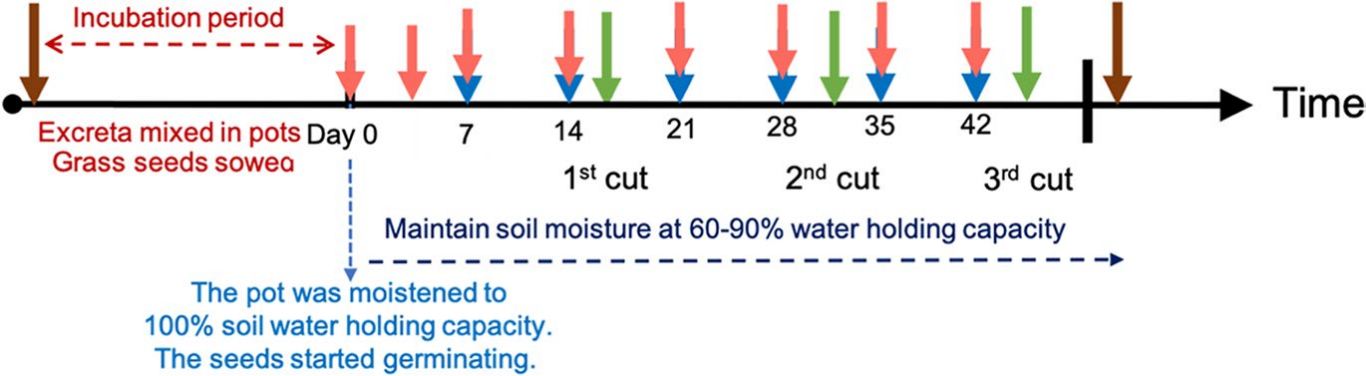
Timeline of the experiment. The arrows in brown, pink, blue and green indicate the timings of sampling soil, sampling soil solution, applying the heavy rain event, and cutting the grass, respectively

After the excreta were applied to the soils, the mixtures in the pots were moistened to 100% water holding capacity (WHC) by sitting the pots in a saucer with ARW and allowing the ARW to be taken up from the bottom of the pot by capillary force for 10 -12 days to equilibrate before the experiment (incubation period). An aliquot of 0.5 g of perennial ryegrass seed was randomly scattered at 1 cm depth below the soil surface of each pot during the incubation period. The day that the seeds germinated was defined as day 0. On day 0, the pots were removed from the water saucer and then placed on the leachate collector. Afterward, the urine was applied to the designated pots at the soil surface. Soil moisture was maintained in the range of 60–90% WHC by weighing the pots and irrigating with ARW every 2–3 d. The 60–90% WHC was the range of moisture that prevented cracking of the soil surface and edges and did not restrict plant growth. A ‘heavy irrigation’ was carried out every 7 d (blue arrows in Fig. 2) by irrigating a volume of 300 mL ARW to each pot in a single day to imitate a heavy rain event (equal to 23 mm precipitation d^−1^). All the leachate from the pot was collected and measured. The results of element leaching were separated into three sample collection periods according to the cutting time of the grass. The grass was cut at the 3.0–4.0 stage of completely developed leaves (about 2 weeks from sowing the seeds). The 3.0–4.0 leaf stage of perennial ryegrass was recommended by Agriculture and Horticulture Development Board of UK as the best time to graze (AHDB 2019). In total, three cuts of grass were carried out during the experiment and the time between each cut was 2 weeks.

### Sample collection and storage

The collected leachate was removed from the collection apparatus, weighed and stored at -18 °C within 24 h. Samples collected within 2 w were bulked to reduce the total number of samples for analysis. The soil solution sample was taken using an embedded soil solution sampler at 2 h after a heavy irrigation event. To sample the soil solution, the protective cap of the sampler was replaced by a blunt fill needle (1.2 mm × 40 mm, with 5 μm filter, BD®) connected to a 10 mL vacutainer tube. Approximately 2 mL soil solution was sampled each time, and normally it took less than 1 h to collect. For those that took more than 1 h, a 25 mL syringe was used instead of the vacutainer tube to collect the sample. The treated soils (with or without feces) collected before the experiment were divided into two parts. One part was kept at -20 °C followed by air drying for later analysis, and the other part was added to the pot. The grass was cut at 2 cm above the soil surface using scissors with stainless-steel blades. This cutting height was used to simulate the grazing depth of sheep which typically ranges from 1 to 2.5 cm depending on the height of grass (ADAS 2011). The cut grass was stored in paper bags and the fresh weight measured, after which the samples were stored at -20 °C before freeze drying.

### Sample preparation and analysis

#### Total elemental (including Se) content in the grass, soil and leachate

The grass samples were freeze-dried and finely ground with a CT293-Cyclotec grinder followed by acid digestion using microwave digestion (MARS, CEM Corporation, 3100 Smith Farm Road, Matthews, NC, USA). A sample of 0.25 g-DM was loaded into a Teflon vessel and 3 mL concentrated nitric acid (HNO_3_) was added and left for 60 min to pre-digest and prevent caking. Following the pre-digestion, an aliquot of 3 mL ultra-pure water (18 MΩ) and 2 mL hydrogen peroxide (H_2_O_2_) were added into the tube and shaken gently. Afterward, the tubes were capped with Teflon lids put into insulation sleeves, and loaded onto the microwave system. The details of the digestion program are shown in Table S4. After the program finished, each of the digested samples were washed into a 50 mL Greiner tube and made up to 50 mL using the 18 MΩ water prior to analysis.

To digest the soil samples, 0.25 g-DM of soil was weighed into a Pyrex® test tube for each sample. A volume of 5 mL aqua regia (4 mL HCl and 1 mL HNO_3_) was then added into each tube. The tubes were placed in a Carbolite® heating block to digest the soil samples. The digestion program is described in Table S5. After the digestion was finished and the heating block was cooled, a volume of 5 mL 25% HNO_3_ was added to each tube, and the heating block reheated to 80 °C and the temperature maintained for 1 h. Afterward, the total volume was made up to 25 mL with ultra-pure water (18 MΩ) prior to analysis. Samples of leachate were defrosted and filtered using 0.45 μm syringe filter and acidified in 5% (v/v) HNO_3_ prior to analysis.

Elemental abundances were measured using ICP-MS (Perkin Elmer® NexION 300X) or ICP-OES (Perkin Elmer® Optima 7300DV and Agilent® 5900 SVDV). The ICP-MS settings were: sample loop size = 1 mL; nebulizer gas flow = 0.91 L min^−1^; auxiliary gas flow = 1.2 L min^−1^; plasma flow = 18 L min^−1^; radio frequency (RF) power = 1600 Watts, kinetic energy discrimination (KED) mode at 3 mL min^−1^ He. The ICP-OES settings were: sample uptake = 1 mL min^−1^; nebulizer gas flow = 0.7 L min^−1^; auxiliary gas flow = 0.3 L min^−1^; plasma flow = 17 L min^−1^; RF power = 1400 Watts.

The ICP-OES was used for concentrations above ca. 50 μg L^−1^ in solution, and the ICP-MS below ca. 50 μg L^−1^. The isotope mass and wavelength used and the estimated detection limit of elements in the ICP-MS and ICP-OES are shown in Table S6.

#### Soil extractable Se, S and P

The soil extractable Se and S were determined using two different P solutions (0.016 M KH_2_PO_4_ (pH = 4.8) and 0.016 M P-buffer (NaH_2_PO_4_/Na_2_HPO_4_, pH = 7.5)). KH_2_PO_4_ is an established reagent for extracting plant-available S (Zhao and McGrath 1994). Due to the chemical similarity between SO_4_^2−^ and SeO_4_^2−^, the reagent is used to extract Se from soil (Stroud et al. 2010). The use of P-buffer in this study is a new attempt to investigate the effect of a different pH environment on Se sorption in the soil. A 5 g-DM soil sample was weighed into a 50 mL sample tube followed by adding 25 mL of one of the P solutions and extracted for 1 h at 25 °C. After extraction, the extracts were filtered through Whatman No.42 filter papers. The supernatants were acidified in 5% HNO_3_ (v/v) before analysis of total Se and S in the extracts using ICP-OES or ICP-MS. The analysis of soil extractable P followed the method of Olsen et al. (1954).

#### Soil solution pH

A pH electrode (InLad Micro, Mettler Toledo®) connected to the pH/ORP meter (Seven2Go, Mettler Toledo®) was calibrated using standard solutions of pH 4, pH 7, and pH 11. After the calibration, the electrodes were inserted directly into the vacutainer tube to take the measurement. The pH of the soil solution samples were measured within 24 h of sample collection.

## Calculations

The Se input contribution from soil and excreta and from irrigation water was calculated using Eqs. 1 and 2, respectively. In Eq. 1, the total dry matter (DM) did not include the DM contribution from urine application. The DM inputs from 70 mL urine from sheep given organic or inorganic mineral supplements were ca. 0.96 ± 0.005 g and 1.28 ± 0.020 g, respectively, which were negligible compared with the DM input from soil and feces (Table 4).

1 $${In}_{s+E}={C}_{S+E}\times {W}_{S+E}$$

In Eq. 1, In_S+E_ = total input of Se from soil and excreta (μg), C_S+E_ = the concentration of Se in the mixture of soil and excreta (μg kg^−1^), and W_S+E_ = total weight (kg-DM) of the mixture of soil and excreta in each pot.

2 $${In}_{w}={C}_{w}\times {V}_{w}$$

In Eq. 2, In_W_ = total input of Se from the irrigation water (μg), C_w_ = the concentration of Se in the irrigation water (μg L^−1^), and V_W_ = total volume (L) of the irrigation water added to each pot.

The Se accumulation in grass across the three cuts were calculated using Eq. 3.

3 $${Se}_{T-accum}={C}_{cut1}\times {W}_{cut1}+ {C}_{cut2}\times {W}_{cut2}+ {C}_{cut3}\times {W}_{cut3}$$

In Eq. 3, C_cut1_, C_cut2_, C_cut3_ = the concentrations (μg kg^−1^) of Se in the first, second, or third cuts, respectively, and W_cut1_, W_cut2_, W_cut3_ = the weight of grass (kg-DM) of the first cut, second, or third cuts, respectively.

### Statistical analysis

A factorial ANOVA model (y ~ Time + Excreta type (ET) + Form of supplemental mineral (Form) + Soil + Interactions (ET x Form + ET x Soil + Form x Soil + ET x Form x Soil)) was performed to test the influences of sample collection time and the three main factors and their interaction on the response variables, including grass growth (DM yield), Se uptake by forage and Se leaching. A principal component analysis (PCA) was performed to analyze the difference in grass at different cutting times based on the total nutrient components (including total cadmium (Cd), Fe, molybdenum (Mo), P, S, zinc (Zn), copper (Cu), manganese (Mn), Se and nitrogen (N)) in the grass. A general ANOVA model (y ~ Block + Treatment) followed by Fisher’s LSD test (α = 0.05) were performed to compare the influence of different treatments on the Se accumulation and concentration in perennial ryegrass, the soil solution pH, soil extractable Se and S and the Se loss into leachate. The LSD test was only performed if significant differences (*P* < 0.05) were identified in the ANOVA tests. QQ-plots were performed, and outliers were removed to ensure that the residuals from the ANOVA models followed a normal distribution. All the statistical analyses were performed in R software (R Core Team 2018).

## Results

### ANOVA results of the treatments and cutting time regarding Se uptake by grass and Se loss into leachate

The different forms (organic/inorganic) of the supplemental minerals to the sheep had no significant impact on either the grass dry matter, Se accumulation in perennial ryegrass nor the Se concentration in perennial ryegrass, but did have a significant effect on total Se loss into leachate associated with soil differences (Table 5). Due to the insignificant impact of supplemental mineral form on Se accumulation and concentration in grass, the results were combined to facilitate data interpretation and discussion. Both the grass DM and Se concentration and accumulation in perennial ryegrass were significantly affected by cutting time, excreta type and soil differences, but with no interaction between excreta type and soil differences. The total Se losses into the leachate were significantly different between the different leachate collection times.

**Table 5:** The ANOVA analysis of grass growth and Se in the grass and leachate of different treatments and cutting times

Factors	Grass dry matter	Se accumulation in perennial ryegrass	Se concentration in perennial ryegrass	Total Se loss into the leachate
Cutting time	< 0.001***	< 0.001***	< 0.001***	< 0.004**
Soil	< 0.001***	0.003**	< 0.001***	0.933
Excreta type (ET)	< 0.001***	0.029*	0.7326	0.991
Form of supplemental minerals to sheep (Form)	0.906	0.103	0.1467	0.591
Soil x ET	0.013*	0.283	0.2889	0.144
ET x Form	0.728	0.270	0.5886	0.892
Soil x Form	0.758	0.291	0.8856	0.045*
Soil x ET x Form	0.842	0.424	0.4990	0.910

Symbols ‘*’, ‘**’, ‘***’ indicate statistical significances of ANOVA test at *p*-value < 0.05, < 0.01, < 0.001, respectively

### Se concentration and accumulation in grass of different cuts

**Fig. 3:**
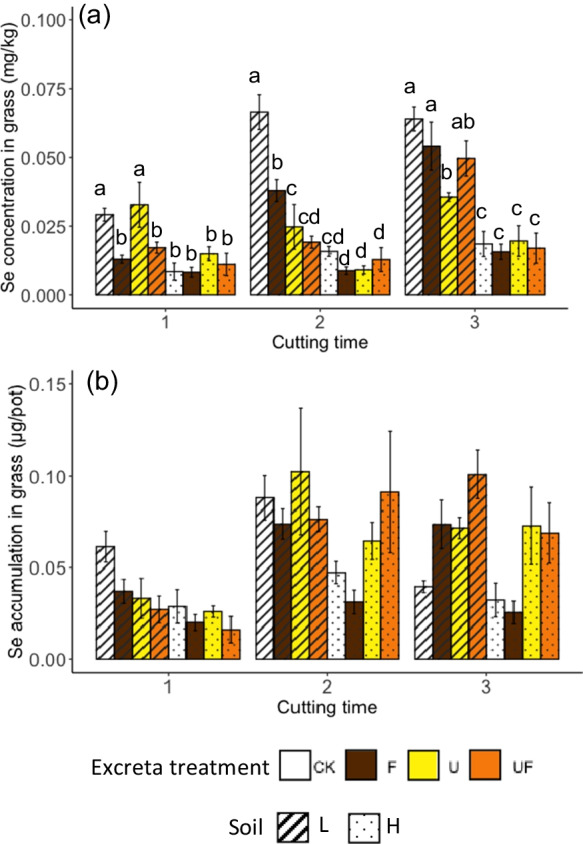
(**a**) Se concentration in the perennial ryegrass (mg kg^−1^) and (**b**) Se accumulation in the perennial ryegrass (μg/pot) of different treatments and of different cut. In the figure legend, the different colors represent the different excreta treatments, with ‘CK’, ‘F’, ‘U’ and ‘UF’ represent for ‘untreated’ and treated with ‘feces’, ‘urine’ and ‘feces + urine’, respectively; the different pattern represent the different soils that the excreta applied to, with ‘L’ and ‘H’ represent for ‘low OM soil’ and ‘high OM soil’, respectively. The error bars are the standard errors of results (*n* = 4 for CK and *n* = 8 for U, F and UF). The lowercase letters indicate the result of Fisher’s LSD test within the same sample batch. The results were not statistically different between bars labelled with the same letter(s)

With no application of sheep excreta, the Se concentrations in grass grown in the soil of low OM (soil L) were, surprisingly, always higher than the grass grown in soil of high OM (soil H), despite of the higher total soil Se in soil H (Fig. 3a). After the application of sheep excreta, regardless of the excreta type, the concentrations of Se in the second cut of grass grown in soil L significantly decreased, whereas the concentrations of Se in the grass of soil H treated with excreta were not significantly different from the untreated soil. The effect of excreta type on Se concentrations differed between the second and third cut of grass in the arable soil. The application of feces resulted in lower Se concentrations than the untreated soil L at the first cut, whereas at the third cut, only the urine-only treatment resulted in lower Se concentration in grass than the untreated soil L. For grass in soil H, the application of different excreta types had no effect on Se concentrations in grass.

Trends in Se accumulation in perennial ryegrass of the different treatments was different across time (Fig. 3b). In the first cut, soil L with no excreta treatment resulted in the highest Se accumulation in the grass, and soil H with the combined urine and feces treatment resulted in the lowest Se accumulation. No difference in Se accumulation in grass was observed between treatments in the second cut. In the third cut, the excreta treatments in soil L appeared to increase the Se accumulation but only the urine and feces combined reached statistical significance. For soil H at the third cut, none of the excreta treatments resulted in significant difference in Se accumulation in grass compared to the untreated soil. However, the urine treatment either with or without feces appeared to increase the Se uptake more than the treatment with feces only.

**Fig. 4:**
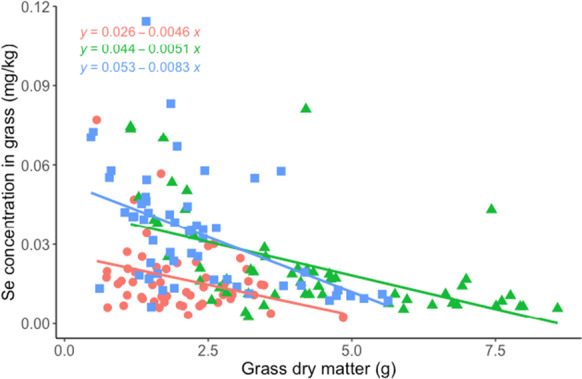
Se concentrations in perennial ryegrass (mg kg^−1^) with grass growth (g-DM) for all treatments. The lines indicate the result of linear regressions for each cut

**Fig. 5:**
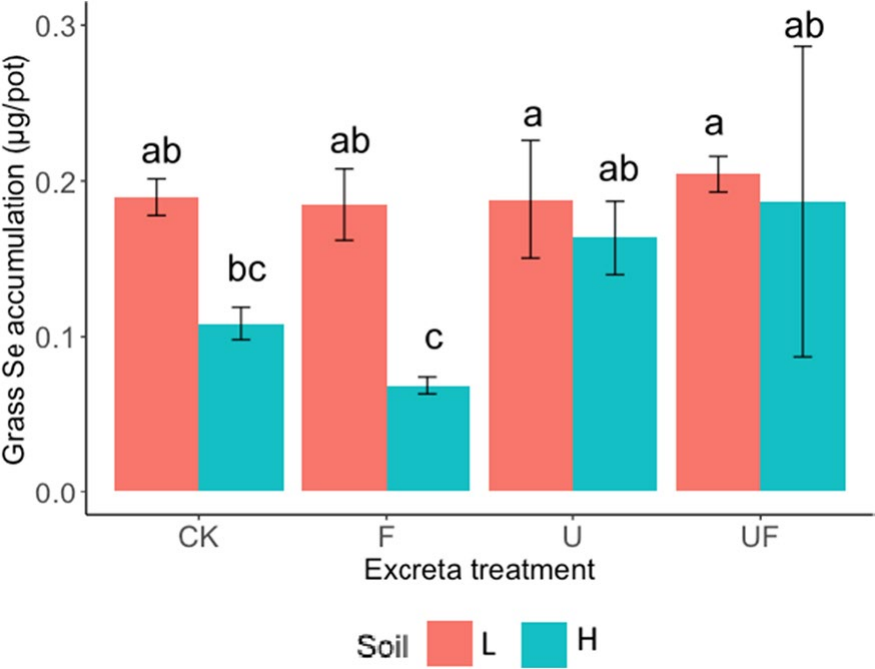
Total Se accumulation in perennial ryegrass (${Se}_{T-accum}$ in μg/pot) throughout the three cutting events. In the figure legend, the different colors represent the different excreta treatments, with ‘CK’, ‘F’, ‘U’ and ‘UF’ represent for ‘untreated’ and treated with ‘feces’, ‘urine’ and ‘feces + urine’, respectively; the different pattern represent the different soils that the excreta applied to, with ‘L’ and ‘H’ represent for ‘low OM soil’ and ‘high OM soil’, respectively. The error bars are the standard errors of the results (*n* = 4 for CK, and *n* = 8 for F, U and UF). Treatment symbols are defined in Table 3. The lowercase letters indicate the result of Fisher’s LSD test comparing the treatments of different excreta and soils. The results were not statistically different between bars labelled with the same letter(s)

For all treatments, the concentration of Se was diluted by the growth of grass, as indicated by the negative slopes of the linear regressions for each cut, which represent the change of Se concentration per unit of biomass increase of grass of all treatments (Fig. 4). The grass grown in the two different soils had significantly different total Se accumulation across three cuts (${Se}_{T-accum}$) when the soils were treated with sheep excreta (Fig. 5). There was no significant difference related to excreta treatment in ${Se}_{T-accum}$ from soil L, whereas in soil H, the treatments containing urine (with or without feces) resulted in higher ${Se}_{T-accum}$ in grass than the treatment with feces only.

### Soil extractable Se, S and P and pH in soil solution

**Fig. 6:**
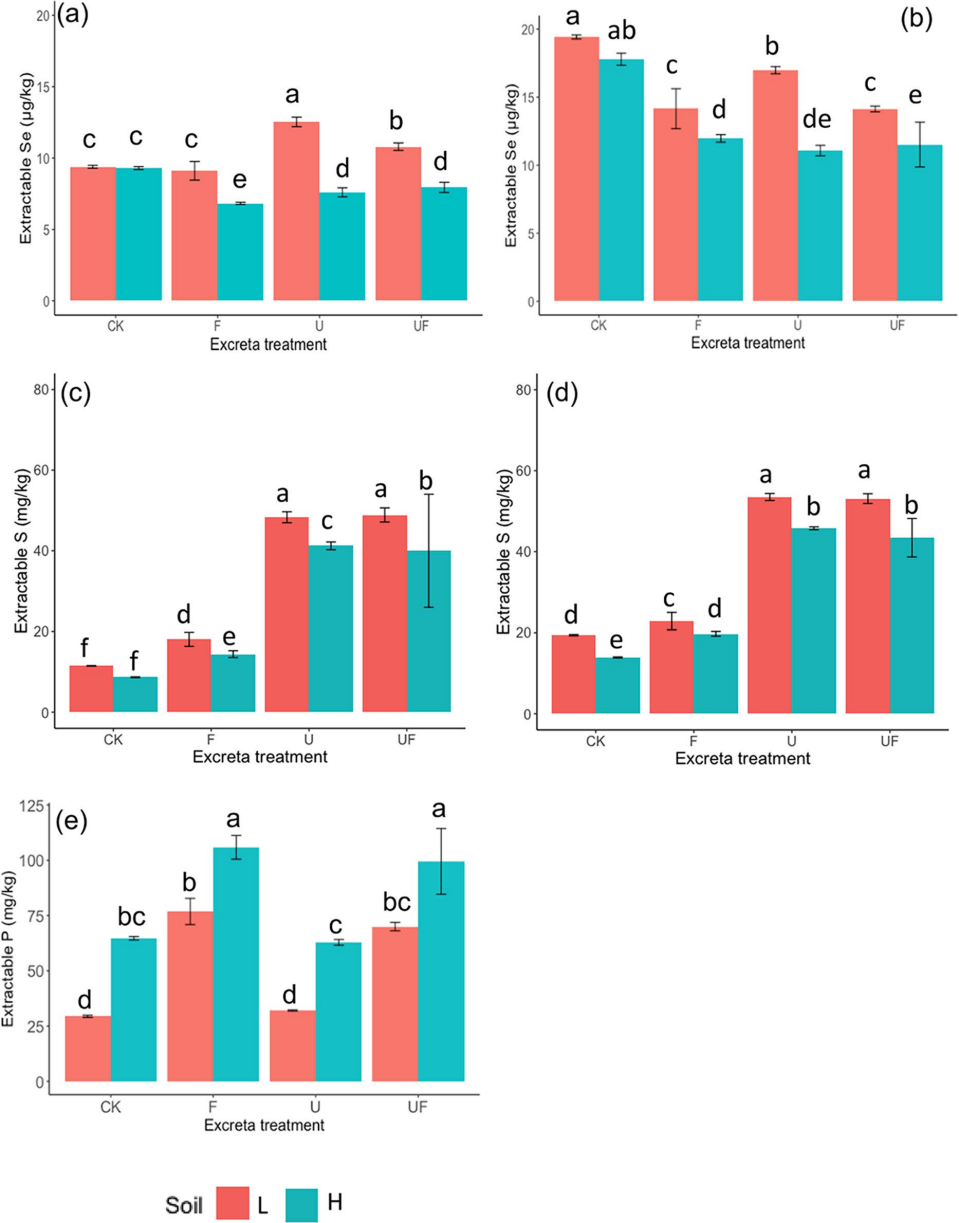
The soil extractable (**a**) Se using MKP solution, (**b**) Se using PB solution, (**c**) S using MKP solution, and (**d**) S using PB solution (**e**) Olsen-P. In the figure legend, the different colors represent the different excreta treatments, with ‘CK’, ‘F’, ‘U’ and ‘UF’ represent for ‘untreated’ and treated with ‘feces’, ‘urine’ and ‘feces + urine’, respectively; the different pattern represent the different soils that the excreta applied to, with ‘L’ and ‘H’ represent for ‘low OM soil’ and ‘high OM soil’, respectively. The error bars are the standard errors of the extraction results (*n* = 3). Treatment symbols are defined in Table 3. The lowercase letters indicate the result of Fisher’s LSD test within the same extraction method. The results were not statistically different between bars labelled with the same letter(s)

In both the P-buffer (PB) and KH_2_PO_4_ (MKP) extractions, the extractable Se of the untreated (no excreta applied) soil L was not significantly different from that of the untreated soil H (Fig. 6). The extraction using MKP (with lower pH), as expected, resulted in lower extractable Se than the extraction using PB extraction (with higher pH). In the MKP extraction, the sheep excreta treatment in soil H had lower extractable Se than the untreated soil H. However, the MKP-extractable Se of the treated soil L was either equal or higher that of the untreated soil L (Fig. 6a). In the PB extraction, the sheep excreta treatments, regardless of the excreta type, significantly lowered the soil extractable Se (Fig. 6b). On the other hand, the difference of soil extractable S across treatments between the two extractions of different pH was relatively small. In both extractions, the extractable S concentrations significantly increased after the application of sheep excreta in both soils (Fig. 6c,d). The treatments of urine (U and UF) resulted in much higher extractable S than the treatments with feces only (F). The extractable S in soil L was higher than that in soil H, except for the untreated (CK) group in the MKP extraction. The soil extractable P was significantly higher in soil H regardless of the excreta treatments (Fig. 6e). The application of feces increased the extractable P in both soils, whereas the application of urine did not have a significant impact on the soil extractable P. The treatments of UF and F resulted in similar soil extractable P.

**Fig. 7:**
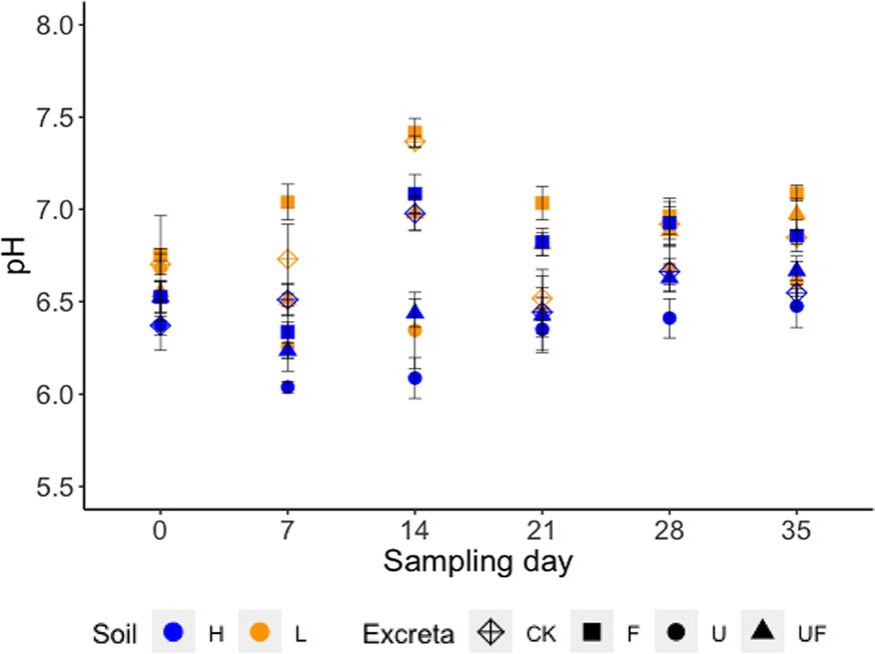
pH variation in the soil solutions through time. Symbols ‘*’, ‘**’, ‘***’ in the color of red and black indicate statistical significances of ANOVA test (within the same sampling day) at p-value < 0.05, < 0.01, < 0.001, for the impact of Soil and Excreta, respectively. In the figure legend, the different colors represent the different excreta treatments, with ‘CK’, ‘F’, ‘U’ and ‘UF’ represent for ‘untreated’ and treated with ‘feces’, ‘urine’ and ‘feces + urine’, respectively; the different pattern represent the different soils that the excreta applied to, with ‘L’ and ‘H’ represent for ‘low OM soil’ and ‘high OM soil’, respectively

Throughout the experiment, the soil solution pH ranged from 6.0 to 8.0 (Fig. 7). Both the soil and the excreta type had a significant impact on the pH of the soil solutions with no significant interaction between them (based on the results of an ANOVA test). The pH values of the different treatments followed the order: F > UF > U and soil L > soil H consistently throughout the experiment. The pH of the original urine and feces were 9.4–9.5 and 8.1, respectively (Table 1). However, after the excreta were applied to soil, the urine lowered the pH and the feces increased the pH of the soil solutions compared to the untreated soils. The soil pH (in water) of the arable and the grassland soil, measured before the experiment, were 6.38 and 6.31, respectively (Table 2).

### Se loss into leachate at different sampling times

**Fig. 8:**
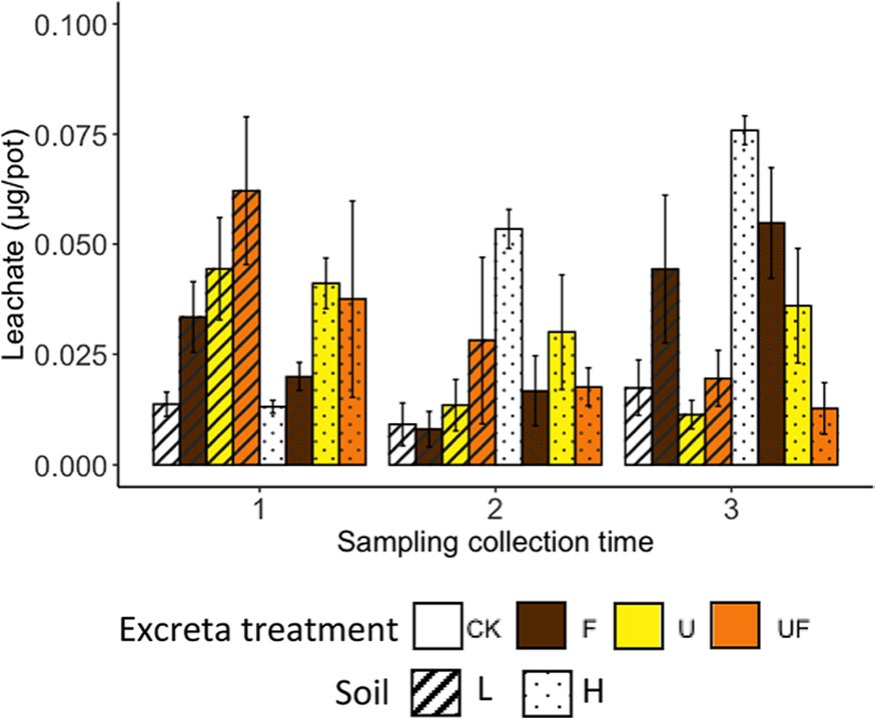
The total Se loss into leachate (μg/pot) at different sampling periods. In the figure legend, the different colors represent the different excreta treatments, with ‘CK’, ‘F’, ‘U’ and ‘UF’ represent for ‘untreated’ and treated with ‘feces’, ‘urine’ and ‘feces + urine’, respectively; the different pattern represent the different soils that the excreta applied to, with ‘L’ and ‘H’ represent for ‘low OM soil’ and ‘high OM soil’, respectively. The error bars are the standard errors of results (*n* = 4 for CK and *n* = 8 for U, F and UF). Treatment symbols are defined in Table 3. The lowercase letters indicate the result of Fisher’s LSD test within the same sampling period. The results were not statistically different between bars labelled with the same letter(s)

In the first sampling, leachate collected from the L-U and L-UF treatments had significantly higher total Se concentrations than the untreated soil L (L-CK) leachate, whereas there was no significant difference between treatments in soil H (Fig. 8). In the second sampling period, there was no difference between treatments. In the third sampling period, the untreated soil H (H-CK) had the highest leachate Se concentration and the treatments with urine (H-U and H-UF) had significantly lower Se in the leachates compared to H-CK. There was no difference in leachate Se concentrations between the treatments of soil L at the third sampling time.

## Discussion

### The effect of the chemical form (organic/inorganic) of supplemental minerals on Se uptake by perennial ryegrass and total Se leaching from the soils

In a previous experiment (data unpublished), the chemical form of supplemental minerals to sheep was hypothesized to be significant to the flux of supplemented micronutrients in pasture systems by changing the excretory pathways of the micronutrients in sheep and also the chemical forms of the micronutrients in the excreta. That study showed that the concentrations of Se in the excreta were not significantly different between supplementation with organic or inorganic minerals. However, higher Se concentrations were observed in the more recalcitrant fractions of a sequential extraction of feces for the inorganic mineral supplements than for the organic mineral supplements. The reason for this observation remains unclear. What effect, if any, the different Se partitioning in feces caused by different supplement forms can have on the uptake of Se by grass and the leaching of Se from soil was explored in the present study.

Results of the pot experiment indicate that the impact of the form of supplemental minerals on Se accumulation and concentration in grass was not significant (Table 5). For Se leaching, although the effect of the interaction between soil and supplement form was significant (*P* = 0.045, Table 5), when the time effect was not included in the ANOVA test (Table S7), the effect of supplement form (either with or without interaction with other factors) on total Se leaching was not significant. This result implies that although the form of mineral supplements given to sheep could affect Se leaching, compared to other factors, i.e. time, soil and excreta type, the impact of supplement form was minor. Therefore, the following discussion focuses on the impact of other significant factors with the data for the different forms (O vs. I) of mineral supplements given to sheep combined.

### Sampling time is a significant factor in Se uptake by grass and leaching

Sample collection time was an important factor to the investigation of Se accumulation and concentration in the grass and Se loss into leachate (Table 5). Except for the first cut, Se concentrations in the grass were higher in soil L than that in soil H, and higher when no excreta was applied (Fig. 3a). In the evaluation of the dilution effect on Se concentration in plants caused by their growth (increase in biomass) (Fig. 4), the slope of the regression line of Cut1 was similar to Cut2 and Cut3, whereas the intercept of the y axis of Cut1 was significantly lower than that of Cut2 and Cut3, meaning that if the grass of Cut 1 were of the same mass as Cut2 and Cut3, the Se concentration in the grass of Cut 1 was lower regardless of the treatments. This result implies that the nutrient uptake and utilization in the grass of Cut1 could have been different from the grass of Cut2 and Cut3, which might be reflected by a supplemented result of a principal component analysis (PCA) (Fig. S3). The PCA analysis, using the elemental composition of grass as the category components, showed that the grass of Cut1 was significantly different from Cut2 and Cu3 (Fig. S3(a)). The grass grew using the nutrients from both the seeds and the regrowth afterward. The nutrient composition of grass growing from seeds can be different from that growing from plants with more established roots (Fulkerson et al. 1998). The grass at Cut2 and Cut3 could have utilized nutrients differently for regrowth from Cut1, which used the nutrients dominantly from the seeds. The density of grass roots at different times might also explain the significantly higher Se leaching during the growing of Cut1 grass compared to the Cut2 grass (*P* < 0.01, Cut1 ≥ Cut3 > Cut2). A greater biomass of plant roots should generally result in less nutrient loss through leaching (Lehmann and Schroth 2003). Indeed, the change of total Se in the leachate through the sampling time could also related to the mobility of Se in the soil and the irrigation (how much water went through the soil column). Therefore, the Se leaching could be the result of multiple factors and the impact of root growth could be just one of them.

### The effects of excreta on dilution of Se concentration in perennial ryegrass during growth

Animal excreta is a commonly used organic fertilizer to add nutrients and organic matter to agricultural soils (Kao et al. 2020). The addition of different types of excreta influenced grass growth (DM yield) (Table 5), which, via the dilution effect, may have partly contributed to the observed difference in Se concentrations in grass of different excreta treatments (Fig. 4). Although time is an important factor in the Se concentration in grass (Table 5), the Se concentrations in the grass of different cuts were all below the concentration requirement of ruminants (0.3, 0.1 and 0.2 mg kg^−1^ for dairy cattle, beef cattle and sheep, respectively, (Kao et al. 2020)). Therefore, a single application of excreta was not able to raise the Se concentrations to the adequate level for ruminant nutrition in the experiments. Although a single dose was unable to raise the level of Se in perennial ryegrass to requirements, the impact of repeating dosing, as would occur in grazing systems, was not investigated here. Instead of raising the Se concentrations in the grass, excreta treatments lowered or had no effect on the concentrations compared with the untreated grass (Fig. 3a). The application of excreta had a greater effect on lowering the Se concentration in grass grown in soil L than in soil H (Fig. 3a), possibly because the Se concentrations of grass grown in the untreated soil H were already low and the Se concentrations of the analytes were close to the detection limit of ICP-MS (equivalent to 0.01 mg kg^−1^ in grass samples). When viewed ${Se}_{T-accum}$, Se uptake was still lower in soil H than in soil L (Fig. 5), which included the effect of approximately two times greater grass DM (Table S8) and also the effect of time. Therefore, the low Se concentration in grass was not solely due to a dilution effect but also resulted from a limited pool of available Se in soil H.

### The impact of soil difference on Se uptake by grass

The Se concentrations (Fig. 3a) and total Se accumulation (${Se}_{T-accum}$, Fig. 5) in the grass of the untreated soil H was always lower than those of the untreated soil L, even though the total soil Se was higher in soil H (865 μg kg^−1^) than in soil L (782 μg kg^−1^). Permanent grassland soils typically have higher soil OM than cultivated soils (Conant et al. 2001). Soil OM is known as a critical factor to the mobility of Se in soils although the sorption of Se might differ with different forms of OM (Kao et al. 2020). In the current study, soil H had significantly higher abundance of soil OM and Fe(III)-(oxyhydr)oxides (35.6 g kg^−1^ and 8200 mg kg^−1^, respectively) than soil L (15.6 g kg^−1^ and 4528 mg kg^−1^, respectively) (Table 2). The Se concentrations in the grass between the two untreated soils might be a reflection of the difference in soil Se sorption. That is, the higher Se sorption driven by higher soil OM and/or redox-active Fe oxides in soil H likely lowered the available Se in the soils, which, in turn, lowered the uptake of Se by the grass. However, the result of the soil extractable Se shows that the extractable Se in the two soils (untreated with excreta) were similar (Fig. 6a,b). Therefore, the differences Se uptake between the two soils cannot be attributed solely to the sorption effect driven by the soil OM and/or the redox-active Fe oxides. It was proposed that the extractable P in soil H, which was higher than that in soil L (Fig. 6e), might have driven a competition between the uptake of PO_4_^3−^ and SeO_3_^2−^, which ultimately lowered the uptake of Se by the grass. This inference will be discussed further in the next section.

### The impact of the applied excreta type on Se accumulation in grass

The greatest Se accumulation in grass was ca. 0.2 μg Se across three cuts (Fig. 5), which was observed in all the treatments of soil L, and in the urine treatments of soil H. Several possible reasons are proposed below that could have driven this result, of which the rationality will be discussed one by one. Firstly, it appears that there was seemingly a ceiling in the total amount of Se that the perennial ryegrass could take up from the soils and, therefore, the input of Se from the excreta did not increase the accumulation of Se. However, this ‘ceiling theory’ could only be true if the amount of *available* Se (represented by the extractable Se) was increased due to the application of excreta, which does not appear to be the case. The Se extracted using the PB solution (pH = 7.5) decreased for the soils with excreta compared to the untreated soils (Fig. 6b), contradicting the ceiling theory. Although Se extracted using MKP solution (pH = 4.8) increased in soil L that were treated with urine, the pH of the extraction environment using PB solution was more similar to the pH environment *in situ* (ca. 6.0–7.5, Fig. 7). Therefore, it is more likely that the available Se in both soil types decreased in response to the application of excreta, regardless of the type. Because of this, the observation seen in Fig. 5 should not be attributed to the ‘ceiling effect’.

Secondly, it may be that the result was due to the difference in availability of soil Se. The contribution of Se input from the feces (12–16 μg Se) was more than 5 times higher than that from the urine (1–2 μg Se). However, the ${Se}_{T-accum}$ of the feces treatments was either not different from (in soil L) or lower than (in soil H) that of the urine treatments (Fig. 5). Some may argue that this is because the Se in the feces was mostly not available to the grass, whereas the Se from the urine was mostly bioavailable. However, as previously noted, extractable Se was not increased by the application of excreta, regardless of the excreta type (except that the extractable Se, using MKP, was higher in soil L that was treated with urine than the untreated), (Fig. 6a,b). Therefore, the differences in Se accumulation in grass between soils with different excreta treatments does not appear to be associated with the Se input.

Thirdly, some may argue that the change of soil pH caused by the application of the excreta could have been playing a part in the result. Fecal treatments resulted in higher soil pH (Fig. 7), which should have *increased* the extractable Se in soils (by limiting Se sorption; (Winkel et al. 2015)) instead of decreasing it (Fig. 6a,b). However, significantly less Se was taken up from soil H when only feces was applied (Fig. 5). Consequently, the difference in Se accumulation in grass from soils with different excreta treatments also cannot be attributed to changes in soil pH caused by the excreta.

Fourthly, the fact that the Se accumulation in grass does not reflect the soil extractable Se leads to the possibility of elemental antagonism, which could decrease the uptake of Se from soils in response to an increase in the abundance of antagonistic element(s). The uptake of SeO_4_^2−^ and SeO_3_^2−^ by grass are subject to competition with SO_4_^2−^ and PO_4_^3−^, respectively, due to the similar electron configuration of the outermost electron shells (Hopper and Parker 1999). SeO_4_^2−^ and SO_4_^2−^ uptake are thought to occur through the same transporters in plants, and the uptake of SeO_3_^2−^ through either passive diffusion that can be inhibited by PO_4_^3−^ (Sors et al. 2005) or active absorption by sharing the transporter with PO_4_^3−^ (Li et al. 2008).

It is unlikely that antagonism by SO_4_^2−^ contributed to the lower Se accumulated by grass in the soil H that was treated only with feces, because extractable S was significantly higher in the treatments with urine applied than in the treatment with only feces applied, and the extractable S was slightly higher in soil L than in the corresponding soil H (Fig. 6c, d). If SO_4_^2−^ antagonism was a major factor, the soils with more extractable S should result in lower ${Se}_{T-accum}$ in grass, the opposite of what was observed (Fig. 5).

Based on the pH and Eh environment of the soils in the current study, the inorganic Se in the soil solution was most likely to be in the form of HSeO_3_^−^. The dominant Se species in the extractable fraction of the feces applied was HSeO_3_^−^ confirmed by HPLC-HG-AFS (details are described in Supplementary Information). SeO_3_^2−^ has a similar electron configuration to PO_4_^3−^ and, therefore, may undergo similar chemical reactions, including surface adsorption and transport by plant roots. Soil extractable PO_4_^3−^ (Fig. 6e) was inversely correlated (R = -0.41) with ${Se}_{T-accum}$ (Fig. S5), which could possibly be attributed to the competition effect between SeO_3_^2−^ and PO_4_^3−^ on the plant uptake. However., for the individual grass cuts, soil extractable PO_4_^3−^ was only significantly correlated with Se concentration and accumulation in the first cut (*P* < 0.001; with R = -0.81 and -0.79, respectively (Fig. S5)). The concentrations of both extractable P and total P in soil H were significantly higher than those in soil L and the application of excreta increased the extractable P, especially in the feces treatment (Fig. 6e and Table 2). The feces applied to the soils contained ca. 12 g kg-DM^−1^ P, and the urine less than 4 mg L^−1^ (Table 1). If the competition effect between SeO_3_^2−^ and PO_4_^3−^ was the dominant factor that affects the Se uptake by grass, the ${Se}_{T-accum}$ of the soil L treated with feces should also be significantly lower than the ${Se}_{T-accum}$ of the untreated soil L. However, this argument was unable to stand based on the results from Fig. 5. Therefore, although PO_4_^3−^ in soil may compete with SeO_3_^2−^ for uptake by grass, it did not appear to be the sole determining factor explaining the variations in Se accumulation in grass across the treatments.

Fifthly, the different excreta treatments did not lead to significantly different loss of Se by leaching (Table 5), and when the leachate samples were bulked across sample times, the combined effects of soil differences and excreta type also became insignificant (Table S7). Therefore, the differences in Se accumulation in grass across the treatments cannot be attributed to Se leaching from the soils either.

### A potential effect of Se speciation on Se accumulation in grass

The speciation of Se in soil strongly affects the mobility and plant availability of Se (Fernández-Martínez and Charlet 2009). Although the speciation of soil Se was not directly analyzed, the Se extracted from the soil using solutions of different pH gives an indication as to the potential species of Se in soils of different treatments. PO_4_^3−^, which has a similar electron configuration to SeO_3_^2−^, has a higher adsorption affinity to oxides than SeO_3_^2−^, SeO_4_^2−^, and SO_4_^2−^ (Balistrieri and Chao 1987), which is why P solutions are used to extract available Se and S. However, Se that is not in the form of SeO_3_^2−^ or SeO_4_^2−^ is unlikely to be exchangeable by PO_4_^3−^ in P solutions. The adsorption of SeO_3_^2−^ or SeO_4_^2−^ to clay minerals, OM and oxides is highly pH-dependent, and is typically greater at lower pH (Winkel et al. 2015). In the current study, the Se concentrations in all the MKP extractions (pH = 4.8) were lower than those in the PB extractions (pH = 7.5). However, this difference was more substantial in the untreated soils (CK) than in the soils treated with urine and/or feces (Fig. 6a.b), which may indicate different Se speciation between the untreated soils and the soils treated with excreta. The application of excreta may have altered the Se speciation and, in turn, altered the availability of Se to the grass.

The speciation of Se, which affects Se mobility and bioavailability, is strongly affected by microbial activities (Fernández-Martínez and Charlet 2009). Alemi et al. (1991) reported that the predominant transformation of SeO_4_^2−^, in an oxic C-enriched soil environment, was microbe-driven reduction of SeO_4_^2−^ to comparatively immobile forms, such as SeO_3_^2−^ and elemental Se. In the excreta treatments, the urine and/or feces provided both carbon and nutrients for microbial metabolism, which could have increased microbial reduction of SeO_4_^2−^ in the soils.

Fernández-Martínez and Charlet (2009) proposed that there are two types of Se reduction that alter the Se species in the environment: dissimilatory reduction and assimilatory reduction, and both are driven by microorganisms. In dissimilatory reduction, microorganisms use the oxidized SeO_4_^2−^ or SeO_3_^2−^ as the terminal electron acceptors for respiration, in the cell membrane, and oxidize organic carbon or reduced S (produced by sulfate-reducing bacteria), producing more reduced forms of Se, such as SeO_3_^2−^ or S^0^. In assimilatory reduction, inorganic Se is transported through the cell membrane into the microbial cell and then reduced during biosynthesis of organic Se compounds, such as the organic acids Se-Met and Se-Cys. Microbial dissimilatory and assimilatory reduction can happen at the same time. For example, some lactic acid bacteria (LAB) used for silage inoculation, can use SeO_3_^2−^ to form Se-Cys up to biological limits and then preferentially convert SeO_3_^2−^ to nano-Se(0) as means of detoxification (Lee et al. 2019b). The synthesis of Se (0) nanoparticles by a filamentous bacterium, isolated from a seleniferous soil, was also observed to happen inside the cells (Tan et al. 2016).

Either microbial reduction pathway could result in lower bioavailability of Se to plants. Although the assimilatory microbial reduction products, Se-Met and Se-Cys, are known to be plant-available and can be taken up by plants more efficiently than inorganic forms of Se (Kikkert and Berkelaar 2013), to increase Se uptake by grass, these compounds would need to be released into the soil environment, rather than incorporated further into proteins in the microbial cells, which would not be readily available to plants. Elemental selenium, Se^0^, the product resulted from a dissimilatory or a assimilatory reduction of SeO_3_^2−^, is substantially less available to plants than SeO_3_^2−^ and SeO_4_^2−^ (Mayland et al. 1991) as it is insoluble (Fernández-Martínez and Charlet 2009) and precipitates out of solution as solid nanoparticles when produced by microorganisms (e.g. Tan et al. 2016). SeO_3_^2−^, which is produced by dissimilatory microbial reduction of SeO_4_^2−^, sorbs more strongly onto Fe oxides than SeO_4_^2−^ (about 2 times higher at pH 7.5 in sorption experiments onto goethite or hematite, Rovira et al. 2008) decreasing its availability for uptake by plants.

Both soil OM and feces can contribute to microbial activity by providing organic carbon. Although roughly half of soil OM is not bioavailable or is highly resistant to decomposition by microorganisms (e.g., Jenkinson and Rayner 1977), the addition of manure to soils has been shown to increase labile (bioavailable) carbon, microbial biomass, and decomposition (e.g. Dheri and Nazir 2021; Ribeiro et al. 2010). An increase in microbial reduction of Se stimulated by the application of excreta could therefore decrease the uptake of Se by grass via lowering the Se mobility and insolubility (after dissimilatory reduction) or by microbial uptake (assimilatory reduction), hence the significantly lower Se accumulation in grass in the grassland soil treated with feces (Fig. 5).

Here, we propose that the lower Se accumulation in grass grown on soil H, particularly with the feces applied, might be explained by the microbial reduction of Se, which either competes with the plants for accessibility of Se, or transforms the Se into less plant-available species. Alos, the reason why the reduced Se accumulation in grass was particularly significant in soil H might be attributed to its higher amount of sorption sites for Se, i.e., Fe oxides and/or OM. However, the reason why the Se accumulation of the soil H treated with the urine (with or without feces) did not have lower Se accumulation in the grass needs to be further investigated.

The current inference proposed by the authors is consistent with previous studies that also reported decreased Se accumulation in plants after animal excreta application. Fan et al. (2008) found decreased Se concentrations in wheat grains after farmyard manure application. Wang et al. (2016) also showed that a 20-year soil application of organic compost led to lower Se accumulation in wheat and maize (*Zea mays*) compared to all other applications including control, inorganic N, P, K plus organic compost, and inorganic N, P, K application. In Wang et al. (2016), despite resulting in the highest soil Se concentration, the application of organic compost did not bring about correspondingly higher Se in the exchangeable fraction. Instead, higher oxidizable Se (i.e. organic Se) was reported compared to the other treatments, resulting in lower Se availability in the soil. Organically bound Se was also found to be the dominant form in all horizons of three Swedish forest soils reported by Gustafsson and Johnsson (1992). Rapid and substantial Se fixation by OM in acidic soils was also observed by Gustafsson and Johnsson (1992).

## Conclusions

Grown in the two soils of different OM contents (total Se concentrations ranged between 0.78–0.87 mg kg^−1^), the Se concentrations in the three cuts of perennial ryegrass were all lower than the requirement needed for growing lambs. The one-time excreta application did not increase Se accumulation and concentrations in the grass and in some case it was decreased. Consequently, to increase ruminant Se intake, it is easier to supplement the animal directly rather than to apply manure as a soil fertilizer, which can potentially drive Se reduction and further decrease in Se uptake by grass.

The lower Se accumulation in the soil of higher OM content, compared to the soil of lower OM content, was likely attributed with the higher extractable PO_4_^3−^, which could drive a competition for the absorption site with SeO_3_^2−^. It was further inferred that, in the animal excreta treatment, the chemical species of Se was possibly altered by microbial reduction, which produces Se in forms that are less soluble and plant-available and could be precipitated as solids, adsorbed to soil particles, or stored in microbial biomass, which might be the main reason for the generally lower Se uptake in grass of the soil of higher OM with feces treated. However, further research is needed to gain direct evidence for this inference.

## Supplementary Information

Below is the link to the electronic supplementary material.

Supplementary file1 (DOCX 1601 KB)

## Data Availability

The following information and data can be found on the data repository of Rothamsted Research (https://doi.org/10.23637/rothamsted.98883). 1. The consumption of silage, concentrate and mineral supplements of the sheep during the feeding period. 2. Sample collection and preparation methods of urine and feces for trace element analysis. 3. Total trace element analytical results of the urine and feces used in the current study. The dataset generated and analyzed during the current pot experiment is available from the Rothamsted Research data repository (10.23637/rothamsted.98v24).
