# Supplementary material for: The uptake of selenium by perennial ryegrass in soils of different organic matter contents receiving sheep excreta
Source: Plant Soil. 2023 Feb 2;486(1-2):639–59. doi: 10.1007/s11104-023-05898-8 (PMC10220126; doi:10.1007/s11104-023-05898-8)
Supplement: Supplementary file 1 — Supplementary file1 (DOCX 1601 KB) [file 11104_2023_5898_MOESM1_ESM.docx]

**Supplementary Information**

**Title**
The uptake of selenium by perennial ryegrass in soils of different organic matter contents receiving sheep excreta

**Author information**

P.-T. Kao^a*^, H.L. Buss^b^, S.P. McGrath^c^, T. Darch^a^, H. Warren^d^, M.R.F. Lee^e^

^a^ Rothamsted Research, North Wyke, Okehampton, Devon, EX20 2SB, UK

^b^ University of Bristol, School of Earth Sciences, Bristol, BS8 1RJ, UK

^c^ Rothamsted Research, Harpenden, Hertfordshire, AL5 2JQ, UK

^d^ Alltech Bioscience Centre, Sarney, Summerhill Road, Dunboyne, Co. Meath, Ireland

^e^ Harper Adams University, Newport, Shropshire, TF10 8NB, UK

**^*^Corresponding author**: Pei-Tzu Kao. email: [b01601029@gmail.com](mailto:b01601029@gmail.com)

**Author ORCIDs**

Pei-Tzu Kao: <https://orcid.org/0000-0002-1380-7781>

Heather L. Buss: <https://orcid.org/0000-0002-1852-3657>

Steve McGrath: https://orcid.org/0000-0003-0952-8947

Tegan Darch: <https://orcid.org/0000-0003-2367-043X>

Helen E. Warren: https://orcid.org/0000-0002-5952-9748

Michael R.F. Lee: <https://orcid.org/0000-0001-7451-5611>

**A mini study to determination the application rate of urine and feces in the current pot experiment**

**Introduction**

To determine the appropriate application rate of feces and urine in the pot experiment, two approaches were trialled to determine the best approach for the main experiment: method 1 – to assess the size of excretion events from grazing fields at North Wyke, Rothamsted Research, UK; method 2 – to rely on the literature from previous grazing experiments. In this preliminary test, both methods were applied to compare the difference and the application rate of feces and urine for the pot experiment were decided accordingly.

**Materials and methods**

*Field fecal sample collection and density calculation*

Photos (n=30) of fresh sheep fecal patches from the farm field at North Wyke were taken. The area of the sheep feces in the same photo were then measured using the software ‘SketchAndCalc™’ developed by iCalc®. The scale of a grid was calibrated by drawing several straight lines on the scale meter and changing the scale of the canvas accordingly. After calibrating the scale, lines were circled around the edge of the fecal patch (**Fig. S1**).


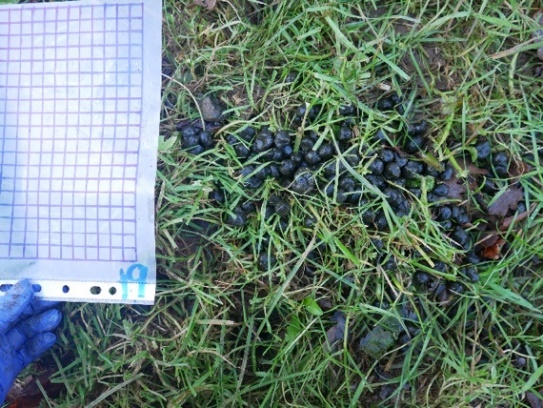

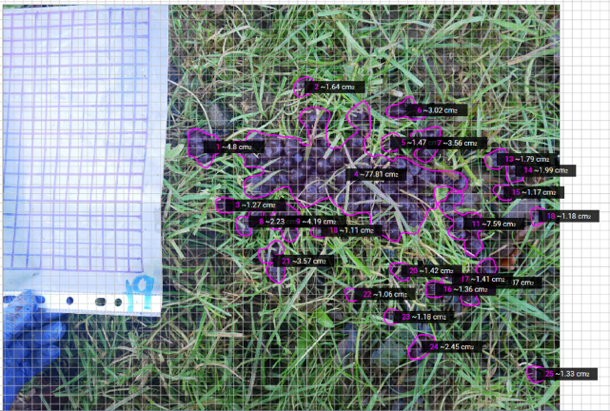


Fig. S1 Sheep feces patch photo taking and area calculation

The area circled was calculated accordingly. At the end, the areas of all the circled fecal patches were summed to give the total area of a fecal patch. Afterwards, the feces of the sample patch were collected by gloved hands into an aluminium-foil box and put in an oven set at 105°C for 48 h. This gave the DM content of the feces of the same patch. The density of feces in field (g cm^-2^) was then calculated accordingly using **Equation S1**:

$D_{F}(faecal patch) =\frac{{In}_{F} (faecal patch)}{A (faecal patch)}$  **(S1)**

$D_{F}(field) =\frac{{In}_{F} (field)}{A(field)}$ (**S2**)

In **Equation S1** **and S2**, D_F_ = fecal density in either a fecal patch or a field, In_F_ = the input of feces in dry matter weight, A= the area included in the measurement (the area of a fecal patch or the area of a field.) It should be remembered that the fecal density calculated using **Equation S1** is higher than the fecal density calculated using **Equation S2**, because in the later calculation, the area of a field includes both areas that are and are not covered by feces.

*Urine density calculation*

Method 1: The urine density (mL cm^-2^) was calculated according to the daily excretion ratio of urine and feces (**Equation S3**). However, this calculation assumes that the daily frequency of urinating and defecating of a sheep are equal, which might overestimate urine density. According to initial statistical results, the data of ratios of the excretion of urine:feces was right skewed (n=23). Therefore, the data was logarithmically transformed. The transformed mean of the ratios was 0.678, which gave the final ratio = 4.764 mL g-DM^-1^ after inversing the data transformation by exponential function.

$D_{U}=D_{F} \times R_{U:F}=D_{F}\times4.764$ **(S3)**

In **Equation S3**, D_U_= urine density in a patch (mL cm^-2^), D_F_= fecal density in either a fecal patch (g-DM cm^-2^), R_U:F_ = the ratio of the excretory amount of urine and feces (mL g-DM^-1^).

Method 2: Calculated the urine density (mL cm^-2^) according to the observed areas of urine patches in field reported by Doak (1952) and the average volume of each urination of sheep reported by Sears et al. (1942).

**Results and discussion**

The investigated fecal densities were averaged after confirming the normality of the data points (**Fig. S2**). The calculated mean via Method 1 of the fecal density of a patch was 0.6 g cm^-2^ (**Table S1**). The average urine density, calculated using **Equation S3** was 2.8 mL cm^-2^ according to the calculated fecal density (**Table S2**). However, it should be remembered that the feces and urine distribution in field calculated using the densities of Method 1 tend to be overestimated. This is because, **Equation S1** excludes the area not covered by feces but still around or within the same fecal patch. If calculated via **Equation S2**, which is used to estimate the average feces input across a field, the fecal density is much lower. For example, White (1960) reported a sheep fecal density in a grassland field of 119 cm^3^ m^-2^ with a sheep density of 157 heads per 100 ha. This fecal density is less than 0.05 g cm^-2^, considering the various moisture content of feces samples.

Method 2 used urine density data reported from the literature. The surface area of soil covered by feces and urine patches at grazing pasture tend to be small and not evenly distributed (Williams and Haynes, 1990). The average covered surface areas reviewed by (Williams and Haynes, 1990) were 30 and 10 cm^2^ for sheep urine and feces, respectively, which were much lowered than the urine covered area (= 290 cm^2^) reported by (Doak, 1952) and the area covered by feces observed via Method 1 in the current study (range = 14 to 134 cm^2^, mean= 43 cm^2^). The observed urine patch area observed by Doak (1952) ranged from 25 to 54 in^2^ (= 161 to 348 cm^2^) and with an average of 45 in^2^ (= 290 cm^2^). Doak (1952) reported a daily volume of urine from sheep varied between 1700 and 3800 mL, with an average of 2900 mL. The volume observed from a sheep experiment at North Wyke was 1760 mL averaged across 24 housed sheep. Doak (1952) used a value of 150 mL as the average volume of each urination event. This number was referred from Sears and Goodall (1942) in which an electrical counting device was operated to record the volume of urination. Using the volume of 150 mL and the mean urinated area 290 cm^2^, it gives a urination density = 0.52 mL cm^-2^, which is five times lower than the density calculated via Method 1.

The calculated feces and urine densities in field can vary widely depending on the method of calculation. In the current pot experiment, if using the feces and urine densities calculated via Method 1, 80 g-DM and 370 mL of feces and urine, respectively, would be applied to each pot. For feces, the 80 g-DM equals to ca. 320 g moist feces at the moisture = 75%. Mixing 320 g moist feces into the soil layer could change the soil structure to a great extent. The change of soil physical structure could become a significant covariate and, therefore, should be prevented as much as possible. Furthermore, the pot experiment was originally designed to mimic the environment of grassland fields as much as possible, the overestimated feces and urine densities could mislead the interpretation of the result. Therefore, in the current pot experiment, the lower urine density = 0.52 mL cm^-2^ calculated via Method 2 was applied, which gives the application amount of 70 mL urine pot^-1^. Calculated using the urine density of 0.52 mL cm^-2^ and the excretion ratio of urine and feces of 4.764 mL g-DM^-1^ (Method 1), a feces density of 0.11 g-DM cm^-2^ (15 g-DM feces pot^-1^) was adopted as the reference of the application amount of feces. However, since the feces was applied ‘into’ the soil instead of being applied on the soil surface, the eventual application amount was adjusted according to the volume of the lysimeter pot.


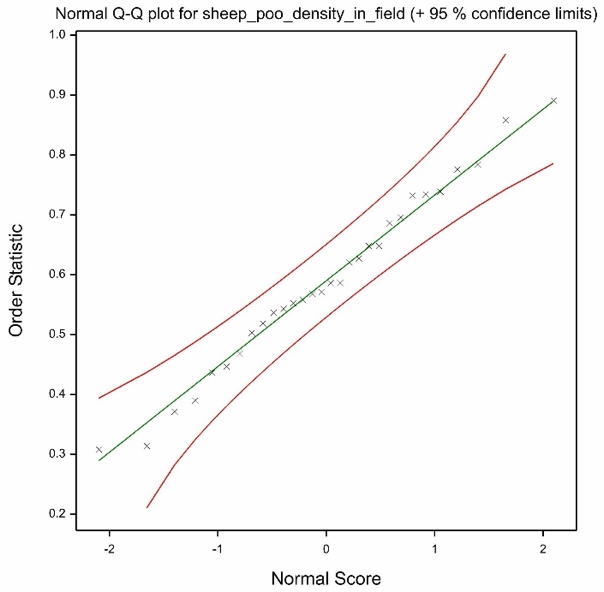

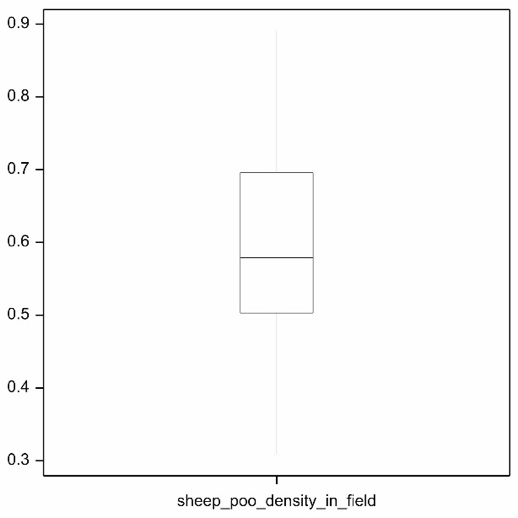


Fig. S2 Normal Q-Q plot (left) and box plot (right) of data of the investigated feces densities (g-DM cm^-2^).

Table S1 Statistical results of the investigated feces densities (g-DM cm^-2^)

| Number of observations = | 30 |
| --- | --- |
| Mean = | 0.590 |
| Median = | 0.579 |
| Minimum = | 0.308 |
| Maximum = | 0.891 |
| Lower quartile = | 0.503 |
| Upper quartile = | 0.696 |

Table S2 Results of calculated urine densities via Method 1

| **Sample number** | **Feces density**  **(g-DM cm^-2^)** | **Calculated urine density (mL cm^-2^)** |
| --- | --- | --- |
| 1 | 0.3 | 1.5 |
| 2 | 0.5 | 2.6 |
| 3 | 0.5 | 2.6 |
| 4 | 0.4 | 1.9 |
| 5 | 0.6 | 2.7 |
| 6 | 0.6 | 2.8 |
| 7 | 0.4 | 2.1 |
| 8 | 0.6 | 2.6 |
| 9 | 0.5 | 2.5 |
| 10 | 0.7 | 3.3 |
| 11 | 0.6 | 2.7 |
| 12 | 0.6 | 3.0 |
| 13 | 0.4 | 2.1 |
| 14 | 0.6 | 2.7 |
| 15 | 0.5 | 2.4 |
| 16 | 0.7 | 3.5 |
| 17 | 0.7 | 3.5 |
| 18 | 0.6 | 2.8 |
| 19 | 0.3 | 1.5 |
| 20 | 0.5 | 2.2 |
| 21 | 0.9 | 4.2 |
| 22 | 0.7 | 3.3 |
| 23 | 0.6 | 3.1 |
| 24 | 0.6 | 3.1 |
| 25 | 0.4 | 1.8 |
| 26 | 0.9 | 4.1 |
| 27 | 0.6 | 3.0 |
| 28 | 0.8 | 3.7 |
| 29 | 0.8 | 3.7 |
| 30 | 0.7 | 3.5 |
| **Average** | **0.6** | **2.8** |

Table S3 The components of 1 L stock solution used for making artificial rainwater

| **Salts** | **Mass (g)** |
| --- | --- |
| NH_4_Cl | 1.385 |
| K_2_SO_4_ | 1.235 |
| HNa_2_PO_4_.2H_2_O | 0.016 |
| FeCl_2_.4H_2_O | 0.089 |
| NaCl | 5.845 |
| CaCl2.2H_2_O | 4.563 |
| NH_4_NO_3_ | 0.560 |
| MgCl_2_.6H_2_O | 4.057 |
| (NH_4_)_2_SO_4_ | 0.319 |

Table S4 Method program of the microwave system used for digesting grass samples

| Power | | Ramp  (minutes) | Temperature  (°C) | Hold  (minutes) |
| --- | --- | --- | --- | --- |
| Max (w) | % |  |  |  |
| 1600 | 100 | 12 | 115 | 1 |
| 1600 | 100 | 8 | 175 | 10 |

Table S5 Method program of Aqua regia digestion of soil in Carbolite heating block

| Ramp  (minutes) | Temperature  (°C) | Hold  (hours) |
| --- | --- | --- |
|  |  |  |
| 1 | 35 | 3 |
| 1 | 60 | 3 |
| 2 | 105 | 1 |
| 2 | 125 | 2 |
| - | 50 | 5 |

**Table S6** The isotope mass and wavelength used and detection limit for each element in the ICP-MS and ICP-OES

| Element | ICP-MS | | ICP-OES | | |
| --- | --- | --- | --- | --- | --- |
|  | Isotope mass (amu) | Detection limit  (μg L^-1^)* | Wavelength (nm) | Detection limit plant digests (mg L^-1^)* | Detection limit soil digests  (mg L^-1^)* |
| Cd | 111 | 0.01 |  |  |  |
| Co | 59 | 0.05 | 228.616 | 0.002 | 0.002 |
| Cu | 63 | 0.03 | 327.393 | 0.005 | 0.006 |
| Fe | 57 | 0.10 | 238.204 | 0.031 | 0.695 |
| Mn | 55 | 0.02 | 257.610 | 0.001 | 0.020 |
| Mo | 95 | 0.02 |  |  |  |
| P |  |  | 213.617 | 0.256 | 0.132 |
| S |  |  | 181.975 | 0.098 | 0.670 |
| Se | 78 | 0.04 |  |  |  |
| Zn |  |  | 206.200 | 0.007 | 0.029 |
| Na |  |  | 589.592 | 0.170 | 0.149 |
| Ca |  |  | 315.887 | 0.027 | 0.283 |

| Factors |  | Elements† | |  | |
| --- | --- | --- | --- | --- | --- |
|  | P (mg) | | S (mg) | | Se (μg) |
| Excreta type (ET) | 0.3180 | | **0.0109 *** | | 0.9933 |
| Supplemental mineral form (Form) | 0.3215 | | 0.1394 | | 0.6453 |
| Soil | **0.0247 *** | | **0.0467 *** | | 0.9427 |
| ET x Form | 0.6112 | | 0.5205 | | 0.9189 |
| ET x Soil | 0.5019 | | 0.7195 | | 0.2458 |
| Form x Soil | **0.0139 *** | | 0.0582 | | 0.0891 |
| ET x Form x Soil | 0.9652 | | 0.2399 | | 0.9325 |

Table S7 Results of ANOVA analysis on the total loss of an element in leachate

†Symbols ‘*’ indicate statistical significances of the ANOVA test at p-value<0.05.


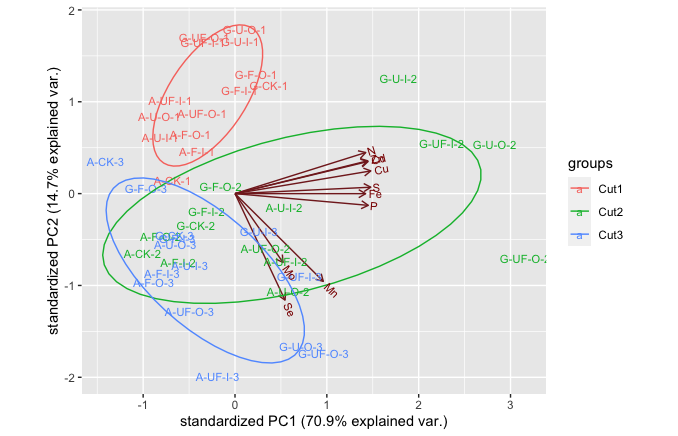

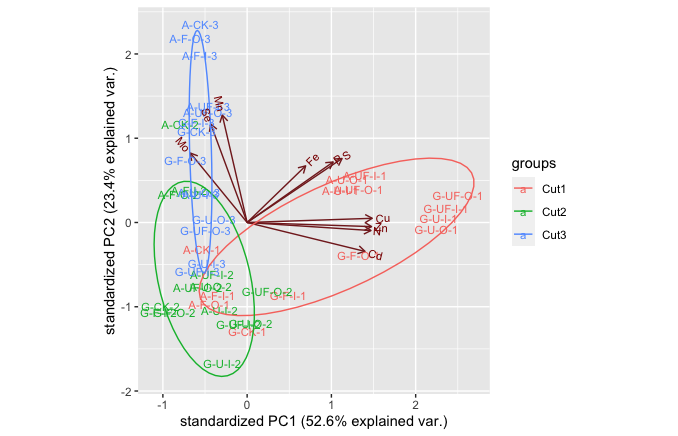


(a)

(b)

Fig. S3 PCA analysis according to either (a) total contents of the elements in grass or (b) concentrations of the elements in grass, grouped by the different cutting time. The data used for producing the figures are Table S9, S10 and S11 for (a) and Table S12, S13 and S14 for (b).

Table S8 Grass dry matter of different cutting times

| Treatments | | First cut | Second cut | Third cut | Total DM |
| --- | --- | --- | --- | --- | --- |
|  |  | (g pot^-1^ ± SE) † | | | |
| A-CK | | 2.12 ± 0.270^bcde^ | 1.33 ± 0.135^e^ | 0.64 ± 0.092^e^ | 4.09 ± 0.164^e^ |
| A-F-I | | 2.73 ± 0.470^abc^ | 2.07 ± 0.096^de^ | 1.41 ± 0.025^cde^ | 6.21 ± 0.550^cd^ |
| A-F-O | | 2.71 ± 0.352^abc^ | 1.85 ± 0.182^e^ | 1.28 ± 0.090^de^ | 5.84 ± 0.242^d^ |
| A-U-I | | 0.95 ± 0.298^f^ | 4.15 ± 0.309^b^ | 2.16 ± 0.274^c^ | 7.26 ± 0.445^bcd^ |
| A-U-O | | 1.20 ± 0.388^ef^ | 4.04 ± 0.301^bc^ | 1.93 ± 0.267^cd^ | 7.17 ± 0.230^bcd^ |
| A-UF-I | | 1.24 ± 0.063^ef^ | 4.09 ± 0.258^b^ | 2.19 ± 0.126^c^ | 7.52 ± 0.353^bc^ |
| A-UF-O | | 1.70 ± 0.466^cdef^ | 4.07 ± 0.209^b^ | 1.87 ± 0.126^cd^ | 7.64 ± 0.545^bc^ |
| G-CK | | 3.74 ± 0.381^a^ | 3.03 ± 0.282^cd^ | 1.68 ± 0.111^cd^ | 8.45 ± 0.670^b^ |
| G-F-I | | 2.87 ± 0.664^ab^ | 3.46 ± 0.766^bc^ | 1.73 ± 0.198^cd^ | 8.06 ± 0.971^b^ |
| G-F-O | | 2.37 ± 0.138^abc^ | 3.52 ± 0.256^bc^ | 1.38 ± 0.287^cde^ | 7.28 ± 0.556_bcd_ |
| G-U-I | | 1.80 ± 0.114^cdef^ | 6.75 ± 0.432^a^ | 3.65 ± 0.414^b^ | 12.2 ± 0.37_a_ |
| G-U-O | | 1.92 ± 0.358^bcdef^ | 7.53 ± 0.384^a^ | 3.94 ± 0.540^ab^ | 13.4 ± 0.24^a^ |
| G-UF-I | | 1.44 ± 0.141^def^ | 6.77 ± 0.480^a^ | 4.53 ± 0.544^c^ | 12.7 ± 0.62^a^ |
| G-UF-O | | 1.47 ± 0.384^def^ | 7.17 ± 0.408^a^ | 4.46 ± 0.491^ab^ | 13.1 ± 0.79^a^ |
|  | P level | | | | |
| Excreta type (ET) | | **<0.001***** | **<0.001***** | **<0.001***** | **<0.001***** |
| Supplemental mineral form (Form) | | 0.7814 | 0.4929 | 0.4556 | 0.8010 |
| Soil | | 0.3020 | **<0.001***** | **<0.001***** | **<0.001***** |
| ET x Form | | 0.5708 | 0.7296 | 0.7983 | 0.2443 |
| ET x Soil | | 0.1818 | **0.0112*** | **<0.001***** | **<0.001***** |
| Form x Soil | | 0.4204 | 0.2214 | 0.6106 | 0.5116 |
| ET x Form x Soil | | 0.9352 | 0.8260 | 0.7022 | 0.4613 |

†Superscript letters indicate the results of Fisher’s LSD test across treatments within one column. Symbols ‘*’and ‘***’ indicate statistical significances of the ANOVA test at p-value<0.05 and <0.001, respectively.

**Speciation analysis of Se in fecal samples using P extractions**

**Introduction**

The aim of this preliminary study was to determine a method for extracting plant-available Se from feces for Se speciation analysis on an HPLC-HG-AFS (LOD = 2.0 μg L^-1^; LOQ = 7.0 μg L^-1^), and also to determine the dominant Se species in the collected fecal samples. Since there is no established method for plant-available Se extraction from fecal samples, the extraction methods adopted in this study refer to the methods that are commonly used in Se extraction in soil (Stroud et al., 2012). Two different P solutions (KH_2_PO_4_ solution and P-buffer (NaH_2_PO_4_/Na_2_HPO_4_) solution) at two different concentrations (0.06 M or 0.016 M) were used to extract fecal samples collected from a previous sheep experiment. Oven-dried or freeze-dried fecal samples were both extracted to compare the potential differences caused by sample drying methods.

**Materials and methods**

Fecal samples before dehydration are not suitable for this analysis because the moisture content in feces dilute the analyte. The fecal samples used in this preliminary test were collected from the organic (O) and inorganic (I) supplementation groups of the sheep experiment. Samples were either oven dried at 80°C or freeze dried. An aliquot of 5 g of dried feces was weighed into a 50 mL polypropylene tube, and then 30 mL of the extractant (0.06 M KH_2_PO_4_ or P-buffer) was added into the tube to make a ratio of feces and extractant= 1:6 (w/v). Stroud et al. (2012a) found that a one-hour extraction of KH_2_PO_4_ solution was sufficient for soil samples, with no additional SeO_3_^2-^ extracted after 3, 6, 24 h of shaking. One hour extraction was also used in other studies of Se species analysis in soils (Martens and Suarez, 1997, Martens and Suarez, 1996). Therefore, in this study, the one-hour approach was adopted. Samples were extracted by mechanical shaking at room temperature for 1 h. After shaking, the samples were centrifuged at rpm= 2500 for 5 min. The supernatant was filtered through a 0.45 μm syringe filter into an analyte tube and analysed immediately in HPLC-HG-AFS.

**Results and discussion**

Both the fecal extracts of I and O groups showed SeO_3_^2-^ was the dominant Se species in the P extracts and no difference was found across extracts of different P solutions (**Fig. S4**). The pH of KH_2_PO_4_ solution was about 4.5, and the pH of the P buffer was 7.4. The results, accordingly, suggest that the pH of the P solutions does not cause difference in the extractable Se species.


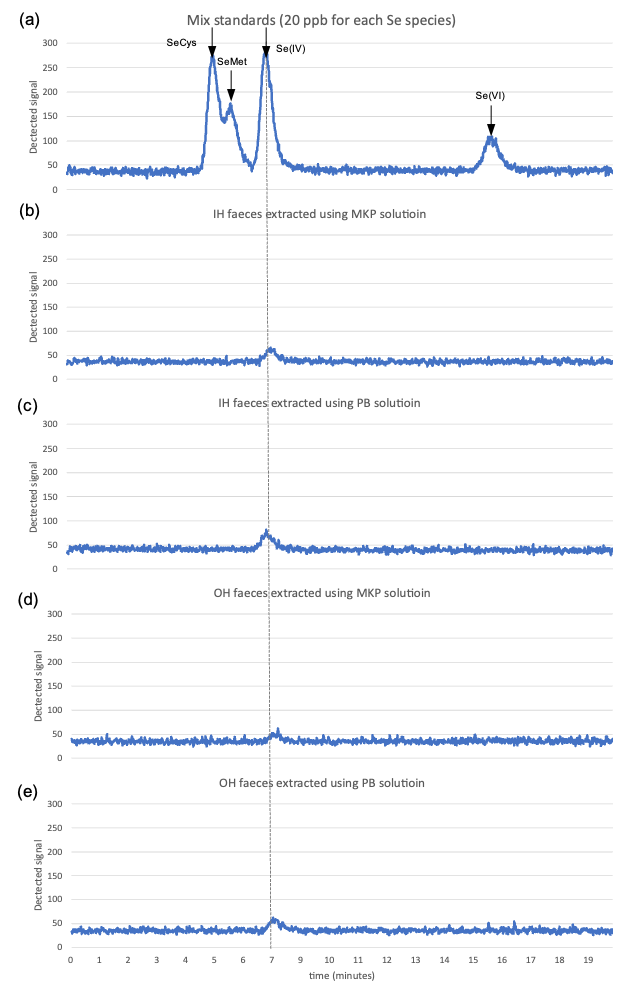


**Fig. S4** HPLC chromatography of Se speciation analysis on feces extracted by different P solutions. The results presented are the chromatographic fingerprints of (a) mixed standard solutions of selenium, and extracted feces of (b) inorganic treatment using MKP (KH_2_PO_4_) solution (c) inorganic treatment using P-buffer (NaH_2_PO_4_/Na_2_HPO_4_) (d) organic treatment using MKP solution (e) inorganic treatment using P-buffer.


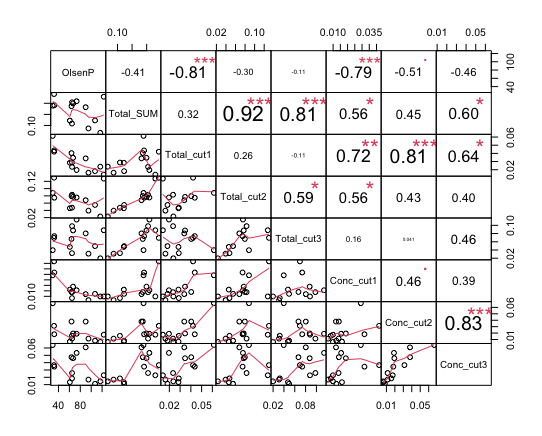


Fig. S5 The correlation map of Olsen-P and Se accumulation or Se concentration in grass of different cuts. Total: total accumulation of Se in grass; Conc: Se concentration in grass; SUM: The total accumulation Se in grass across the three cuts (Se_T-accum_); The star-symbols: ‘***’, ‘**’, ‘*’ represent significant result of Pearson’s correlation test with a p-value <0.001, <0.01, <0.05, respectively.

Table S9 Nutrient accumulation in grass of the first cut

| Treatments | Cd (μg pot^-1^) | Cu (mg pot^-1^) | Fe (mg pot^-1^) | Mn (mg pot^-1^) | Mo (μg pot^-1^) | P (mg pot^-1^) | S (mg pot^-1^) | Se (μg pot^-1^) | Zn (mg pot^-1^) | N (g pot^-1^) |
| --- | --- | --- | --- | --- | --- | --- | --- | --- | --- | --- |
| A-CK | 0.064±0.0068 | 0.011±0.0068 | 0.156±0.0524 | 0.115±0.0157 | 0.915±0.1810 | 6.08±0.795 | 3.90±0.391 | 0.061±0.0085 | 0.034±0.0048 | 3.59±0.255 |
| A-U-O | 0.138±0.0377 | 0.012±0.0337 | 0.089±0.0249 | 0.117±0.0249 | 0.278±0.0694 | 3.44±0.902 | 3.01±0.806 | 0.042±0.0096 | 0.041±0.0112 | 4.49±1.294 |
| A-U-I | 0.130±0.0411 | 0.012±0.0411 | 0.101±0.0400 | 0.146±0.0400 | 0.281±0.1196 | 3.35±1.032 | 3.12±0.745 | 0.044±0.0260 | 0.040±0.0104 | 3.72±1.038 |
| A-F-O | 0.098±0.0106 | 0.016±0.0106 | 0.124±0.0087 | 0.112±0.0087 | 1.345±0.1987 | 8.44±0.864 | 4.46±0.343 | 0.034±0.0081 | 0.049±0.0050 | 4.35±0.128 |
| A-F-I | 0.106±0.0164 | 0.017±0.0164 | 0.121±0.0151 | 0.126±0.0151 | 1.498±0.2860 | 8.27±1.153 | 4.75±0.649 | 0.040±0.0113 | 0.050±0.0054 | 4.57±0.477 |
| A-UF-O | 0.135±0.0226 | 0.017±0.0226 | 0.126±0.0262 | 0.144±0.0262 | 0.735±0.1755 | 7.65±1.798 | 4.21±0.946 | 0.035±0.0138 | 0.067±0.0160 | 5.79±1.230 |
| A-UF-I | 0.119±0.0102 | 0.013±0.0102 | 0.088±0.0072 | 0.146±0.0072 | 0.545±0.0479 | 5.56±0.371 | 3.36±0.274 | 0.020±0.0051 | 0.051±0.0045 | 4.70±0.396 |
| G-CK | 0.231±0.0207 | 0.027±0.0207 | 0.210±0.0297 | 0.160±0.0297 | 1.175±0.1024 | 11.7±1.36 | 5.03±0.594 | 0.029±0.0092 | 0.114±0.0045 | 7.77±0.860 |
| G-U-O | 0.296±0.0587 | 0.023±0.0587 | 0.195±0.0359 | 0.099±0.0359 | 0.476±0.0751 | 8.78±1.336 | 5.29±0.837 | 0.023±0.0047 | 0.086±0.0151 | 8.53±1.558 |
| G-U-I | 0.296±0.0376 | 0.021±0.0376 | 0.237±0.0760 | 0.098±0.0760 | 0.464±0.0471 | 7.82±0.795 | 5.00±0.361 | 0.028±0.0044 | 0.081±0.0075 | 7.81±0.676 |
| G-F-O | 0.267±0.0259 | 0.025±0.0259 | 0.158±0.0165 | 0.109±0.0165 | 1.961±0.1506 | 9.89±0.785 | 5.47±0.411 | 0.024±0.0066 | 0.108±0.0097 | 7.65±0.666 |
| G-F-I | 0.228±0.0333 | 0.023±0.0333 | 0.157±0.0250 | 0.117±0.0250 | 2.212±0.6280 | 9.62±1.430 | 4.93±0.539 | 0.016±0.0059 | 0.095±0.0125 | 6.42±0.763 |
| G-UF-O | 0.208±0.0542 | 0.018±0.0542 | 0.145±0.0268 | 0.083±0.0268 | 0.581±0.1785 | 7.27±1.572 | 4.52±0.952 | 0.014±0.0033 | 0.071±0.0173 | 6.31±1.547 |
| G-UF-I | 0.221±0.0351 | 0.017±0.0351 | 0.134±0.0166 | 0.080±0.0166 | 0.566±0.0903 | 7.32±0.843 | 4.46±0.471 | 0.018±0.0048 | 0.071±0.0091 | 6.21±0.702 |

Table S10 Nutrient accumulation in grass of the second cut

| Treatments | Cd (μg pot^-1^) | Cu (mg pot^-1^) | Fe (mg pot^-1^) | Mn (mg pot^-1^) | Mo (μg pot^-1^) | P (mg pot^-1^) | S (mg pot^-1^) | Se (μg pot^-1^) | Zn (mg pot^-1^) | N (g pot^-1^) |
| --- | --- | --- | --- | --- | --- | --- | --- | --- | --- | --- |
| A-CK | 0.030±0.0035 | 0.007±0.0002 | 0.082±0.0254 | 0.139±0.0130 | 0.920±0.2166 | 4.18±0.183 | 2.76±0.183 | 0.088±0.0121 | 0.023±0.0010 | 1.81±0.110 |
| A-U-O | 0.281±0.0576 | 0.024±0.0032 | 0.236±0.0536 | 0.281±0.0473 | 0.743±0.1569 | 8.89±0.815 | 6.87±0.815 | 0.130±0.0708 | 0.077±0.0122 | 8.22±1.224 |
| A-U-I | 0.294±0.0259 | 0.026±0.0030 | 0.197±0.0123 | 0.308±0.0194 | 0.666±0.0404 | 8.82±0.545 | 7.02±0.545 | 0.075±0.0061 | 0.080±0.0035 | 8.78±0.929 |
| A-F-O | 0.041±0.0069 | 0.009±0.0009 | 0.070±0.0084 | 0.137±0.0143 | 1.77±0.160 | 6.03±0.219 | 3.89±0.219 | 0.068±0.0110 | 0.029±0.0026 | 2.65±0.219 |
| A-F-I | 0.046±0.0040 | 0.011±0.0006 | 0.084±0.0031 | 0.155±0.0240 | 1.93±0.158 | 7.18±0.262 | 4.57±0.262 | 0.080±0.0131 | 0.035±0.0024 | 3.02±0.202 |
| A-UF-O | 0.179±0.0063 | 0.023±0.0006 | 0.175±0.0169 | 0.230±0.0078 | 2.00±0.073 | 13.2±0.17 | 6.07±0.166 | 0.077±0.0092 | 0.073±0.0030 | 6.16±0.286 |
| A-UF-I | 0.227±0.0230 | 0.025±0.0017 | 0.179±0.0103 | 0.301±0.0220 | 1.94±0.120 | 12.9±0.38 | 6.61±0.375 | 0.076±0.0109 | 0.079±0.0049 | 7.33±0.733 |
| G-CK | 0.115±0.0190 | 0.014±0.0016 | 0.098±0.0101 | 0.204±0.0188 | 2.33±0.349 | 7.90±0.831 | 3.40±0.415 | 0.047±0.0060 | 0.055±0.0059 | 4.21±0.460 |
| G-U-O | 0.656±0.0365 | 0.048±0.0027 | 0.488±0.0660 | 0.334±0.0173 | 1.78±0.306 | 19.8±0.66 | 13.2±0.34 | 0.083±0.0152 | 0.168±0.0069 | 17.5±0.86 |
| G-U-I | 0.577±0.0917 | 0.037±0.0029 | 0.370±0.0444 | 0.255±0.0288 | 1.27±0.187 | 15.1±0.80 | 10.3±0.61 | 0.046±0.0046 | 0.138±0.0135 | 13.8±0.74 |
| G-F-O | 0.166±0.0240 | 0.016±0.0008 | 0.167±0.0552 | 0.122±0.0101 | 3.95±0.128 | 9.11±0.527 | 5.25±0.140 | 0.023±0.0087 | 0.059±0.0052 | 4.68±0.330 |
| G-F-I | 0.139±0.0465 | 0.015±0.0030 | 0.116±0.0235 | 0.140±0.0104 | 3.44±0.521 | 8.50±1.685 | 4.67±0.566 | 0.039±0.0089 | 0.052±0.0123 | 4.54±1.078 |
| G-UF-O | 0.696±0.0387 | 0.051±0.0038 | 0.501±0.0699 | 0.334±0.0150 | 3.70±0.550 | 21.1±1.09 | 12.9±0.49 | 0.128±0.0648 | 0.196±0.0163 | 16.8±1.07 |
| G-UF-I | 0.537±0.0652 | 0.041±0.0030 | 0.456±0.0319 | 0.278±0.0369 | 2.92±0.301 | 18.6±1.30 | 11.0±0.93 | 0.055±0.0069 | 0.154±0.0133 | 14.8±1.34 |

Table S11 Nutrient accumulation in grass of the third cut

| Treatments | Cd (μg pot^-1^) | Cu (mg pot^-1^) | Fe (mg pot^-1^) | Mn (mg pot^-1^) | Mo (μg pot^-1^) | P (mg pot^-1^) | S (mg pot^-1^) | Se (μg pot^-1^) | Zn (mg pot^-1^) | N (g pot^-1^) |
| --- | --- | --- | --- | --- | --- | --- | --- | --- | --- | --- |
| A-CK | 0.016±0.0031 | 0.003±0.0005 | 0.063±0.0282 | 0.103±0.0110 | 0.543±0.0502 | 2.20±0.287 | 1.69±0.234 | 0.040±0.0033 | 0.011±0.0015 | 0.89±0.124 |
| A-U-O | 0.092±0.0176 | 0.012±0.0016 | 0.105±0.0150 | 0.237±0.0213 | 0.650±0.1015 | 6.46±0.798 | 3.03±0.279 | 0.069±0.0072 | 0.036±0.0051 | 2.70±0.398 |
| A-U-I | 0.120±0.0169 | 0.013±0.0011 | 0.116±0.0145 | 0.273±0.0139 | 0.826±0.0534 | 7.00±0.440 | 3.13±0.308 | 0.074±0.0101 | 0.041±0.0046 | 3.05±0.401 |
| A-F-O | 0.027±0.0048 | 0.007±0.0004 | 0.101±0.0334 | 0.162±0.0255 | 1.98±0.2951 | 4.74±0.285 | 3.37±0.303 | 0.080±0.0277 | 0.021±0.0018 | 1.83±0.113 |
| A-F-I | 0.029±0.0010 | 0.008±0.0003 | 0.098±0.0087 | 0.189±0.0194 | 2.08±0.1074 | 5.40±0.177 | 3.86±0.164 | 0.067±0.0040 | 0.025±0.0009 | 2.12±0.114 |
| A-UF-O | 0.069±0.0071 | 0.013±0.0007 | 0.105±0.0091 | 0.214±0.0100 | 2.31±0.1108 | 8.21±0.598 | 3.38±0.189 | 0.088±0.0174 | 0.037±0.0024 | 2.81±0.172 |
| A-UF-I | 0.108±0.0122 | 0.015±0.0012 | 0.133±0.0129 | 0.276±0.0184 | 2.50±0.1916 | 9.09±0.576 | 3.65±0.215 | 0.114±0.0196 | 0.047±0.0050 | 3.20±0.193 |
| G-CK | 0.079±0.0058 | 0.008±0.0006 | 0.230±0.0941 | 0.248±0.0098 | 1.93±0.2376 | 4.99±0.243 | 2.29±0.157 | 0.032±0.0093 | 0.033±0.0019 | 2.05±0.146 |
| G-U-O | 0.265±0.0475 | 0.022±0.0027 | 0.264±0.0562 | 0.392±0.0241 | 1.99±0.4367 | 12.9±1.47 | 6.31±0.704 | 0.098±0.0406 | 0.083±0.0116 | 5.50±0.695 |
| G-U-I | 0.211±0.0085 | 0.019±0.0016 | 0.238±0.0293 | 0.330±0.0106 | 1.26±0.1469 | 10.9±0.92 | 5.54±0.493 | 0.048±0.0039 | 0.072±0.0061 | 4.74±0.478 |
| G-F-O | 0.067±0.0163 | 0.007±0.0014 | 0.122±0.0423 | 0.128±0.0223 | 2.71±0.5332 | 4.50±0.913 | 2.48±0.462 | 0.020±0.0040 | 0.025±0.0055 | 1.77±0.357 |
| G-F-I | 0.076±0.0116 | 0.008±0.0011 | 0.231±0.0758 | 0.181±0.0186 | 3.15±0.6120 | 5.48±0.590 | 3.10±0.129 | 0.031±0.0121 | 0.029±0.0047 | 2.13±0.225 |
| G-UF-O | 0.253±0.0209 | 0.026±0.0024 | 0.320±0.0370 | 0.317±0.0219 | 4.25±0.3063 | 14.5±1.28 | 6.86±0.639 | 0.088±0.0314 | 0.094±0.0115 | 6.18±0.625 |
| G-UF-I | 0.244±0.0237 | 0.023±0.0019 | 0.252±0.0202 | 0.319±0.0304 | 3.44±0.3238 | 14.2±1.39 | 6.57±0.585 | 0.050±0.0050 | 0.089±0.0077 | 6.07±0.625 |

Table S12 Micronutrient concentrations in grass of the first cut

| Treatments | Cd (mg kg^-1^) | Cu (mg kg^-1^) | Fe (mg kg^-1^) | Mn (mg kg^-1^) | Mo (mg kg^-1^) | P (mg kg^-1^) | S (mg kg^-1^) | Se (mg kg^-1^) | Zn (mg kg^-1^) | N (% DM) |
| --- | --- | --- | --- | --- | --- | --- | --- | --- | --- | --- |
| A-CK | 0.031±0.0016 | 5.50±0.311 | 75.8±27.35 | 53.9±1.87 | 0.459±0.1189 | 2869±117.8 | 1861±77.1 | 0.029±0.0023 | 16.3±0.97 | 1.74±0.131 |
| A-U-O | 0.125±0.0134 | 10.5±0.57 | 77.7±7.80 | 113±18.5 | 0.253±0.0421 | 3043±304.5 | 2645±196.1 | 0.042±0.0132 | 36.1±3.18 | 3.87±0.153 |
| A-U-I | 0.108±0.0074 | 10.3±0.53 | 81.5±11.97 | 122±11.7 | 0.223±0.0381 | 2788±196.0 | 2674±71.4 | 0.032±0.0109 | 34.5±1.88 | 4.12±0.194 |
| A-F-O | 0.037±0.0030 | 6.12±0.720 | 47.9±6.40 | 40.9±2.45 | 0.494±0.0351 | 3163±154.2 | 1695±137.3 | 0.012±0.0018 | 18.5±0.96 | 1.70±0.143 |
| A-F-I | 0.041±0.0055 | 6.88±1.140 | 46.4±4.37 | 44.8±3.03 | 0.544±0.0354 | 3118±186.0 | 1800±129.7 | 0.014±0.0025 | 19.3±1.93 | 1.78±0.217 |
| A-UF-O | 0.085±0.0077 | 10.4±0.44 | 78.5±9.73 | 88.4±9.62 | 0.441±0.0232 | 4596±166.9 | 2553±108.8 | 0.019±0.0022 | 40.4±1.41 | 3.53±0.176 |
| A-UF-I | 0.096±0.0076 | 10.5±0.43 | 70.5±2.72 | 118±7.9 | 0.438±0.0268 | 4463±87.2 | 2691±91.6 | 0.016±0.0035 | 40.8±2.03 | 3.77±0.152 |
| G-CK | 0.062±0.0037 | 7.24±0.265 | 56.5±7.35 | 42.5±1.56 | 0.317±0.0210 | 3122±157.6 | 1342±59.1 | 0.008±0.0031 | 30.4±1.65 | 2.09±0.185 |
| G-U-O | 0.153±0.0080 | 12.1±0.36 | 101±4.0 | 51.2±2.23 | 0.253±0.0148 | 4660±203.1 | 2808±120.4 | 0.014±0.0046 | 44.9±1.97 | 4.44±0.104 |
| G-U-I | 0.164±0.0139 | 11.8±0.39 | 127±31.2 | 53.8±4.50 | 0.261±0.0295 | 4328±160.2 | 2781±41.8 | 0.016±0.0029 | 45.2±1.30 | 4.33±0.105 |
| G-F-O | 0.113±0.0069 | 10.6±0.52 | 66.3±3.72 | 45.9±2.71 | 0.825±0.0305 | 4160±160.0 | 2306±101.4 | 0.010±0.0025 | 45.7±3.00 | 3.22±0.194 |
| G-F-I | 0.087±0.0171 | 8.47±1.151 | 57.5±5.94 | 42.7±4.71 | 0.750±0.0363 | 3524±324.5 | 1841±205.6 | 0.007±0.0026 | 35.3±4.86 | 2.44±0.404 |
| G-UF-O | 0.144±0.0771 | 12.5±0.37 | 106±15.1 | 56.7±1.57 | 0.380±0.0338 | 5178±302.1 | 3220±172.2 | 0.010±0.0020 | 49.1±1.23 | 4.38±0.103 |
| G-UF-I | 0.151±0.0122 | 11.9±0.39 | 92.7±4.44 | 55.3±2.94 | 0.392±0.0473 | 5075±129.8 | 3098±34.3 | 0.012±0.0027 | 48.8±1.80 | 4.31±0.091 |

Table S13 Micronutrient concentrations in grass of the second cut

| Treatments | Cd (mg kg^-1^) | Cu (mg kg^-1^) | Fe (mg kg^-1^) | Mn (mg kg^-1^) | Mo (mg kg^-1^) | P (mg kg^-1^) | S (mg kg^-1^) | Se (mg kg^-1^) | Zn (mg kg^-1^) | N (% DM) |
| --- | --- | --- | --- | --- | --- | --- | --- | --- | --- | --- |
| A-CK | 0.023±0.0022 | 5.19±0.342 | 58.5±11.37 | 106.8±11.65 | 0.675±0.0967 | 3194±184.6 | 2109±133.2 | 0.067±0.0063 | 17.5±1.32 | 1.38±0.058 |
| A-U-O | 0.069±0.0126 | 5.84±0.504 | 57.4±11.05 | 68.6±8.81 | 0.184±0.0355 | 2208±162.7 | 1687±114.1 | 0.031±0.0167 | 19.0±2.28 | 2.00±0.200 |
| A-U-I | 0.074±0.0130 | 6.35±0.816 | 48.0±3.69 | 75.7±8.06 | 0.162±0.0106 | 2120±145.8 | 1726±191.9 | 0.018±0.0014 | 19.6±1.69 | 2.17±0.323 |
| A-F-O | 0.022±0.0019 | 5.00±0.077 | 37.7±0.92 | 77.4±12.03 | 0.976±0.1104 | 3274±73.7 | 2129±112.7 | 0.038±0.0066 | 15.9±0.23 | 1.44±0.043 |
| A-F-I | 0.022±0.0015 | 5.41±0.239 | 40.5±1.69 | 74.0±9.62 | 0.931±0.0500 | 3465±172.3 | 2206±70.2 | 0.038±0.0055 | 16.7±0.67 | 1.46±0.057 |
| A-UF-O | 0.044±0.0031 | 5.67±0.181 | 43.3±4.32 | 57.0±3.24 | 0.495±0.0258 | 3238±104.5 | 1499±52.4 | 0.019±0.0030 | 18.1±0.72 | 1.52±0.051 |
| A-UF-I | 0.056±0.0051 | 6.17±0.313 | 44.0±1.02 | 74.5±6.82 | 0.481±0.0436 | 3154±45.5 | 1626±87.3 | 0.019±0.0036 | 19.4±0.82 | 1.79±0.135 |
| G-CK | 0.037±0.0028 | 4.54±0.108 | 32.1±0.39 | 68.0±5.76 | 0.756±0.0472 | 2596±49.2 | 1115±68.5 | 0.016±0.0018 | 18.0±0.32 | 1.38±0.038 |
| G-U-O | 0.088±0.0080 | 6.49±0.642 | 66.2±11.36 | 44.7±3.24 | 0.240±0.0454 | 2661±191.0 | 1776±110.3 | 0.011±0.0025 | 22.7±1.88 | 2.36±0.220 |
| G-U-I | 0.084±0.0080 | 5.45±0.093 | 54.3±3.10 | 37.6±2.36 | 0.185±0.0159 | 2252±84.5 | 1530±8.7 | 0.007±0.0005 | 20.3±0.70 | 2.05±0.065 |
| G-F-O | 0.047±0.0045 | 4.64±0.173 | 47.7±15.93 | 35.4±4.08 | 1.132±0.0463 | 2595±41.9 | 1506±73.7 | 0.006±0.0019 | 16.6±0.61 | 1.33±0.019 |
| G-F-I | 0.038±0.0042 | 4.39±0.111 | 34.0±2.03 | 44.1±5.76 | 1.031±0.0704 | 2489±51.0 | 1421±110.1 | 0.011±0.0010 | 15.0±0.66 | 1.30±0.022 |
| G-UF-O | 0.098±0.0071 | 7.19±0.705 | 69.6±7.95 | 46.7±1.03 | 0.509±0.0583 | 2966±211.9 | 1824±153.4 | 0.017±0.0086 | 27.6±2.89 | 2.38±0.273 |
| G-UF-I | 0.079±0.0039 | 6.02±0.075 | 68.1±5.52 | 40.5±2.71 | 0.428±0.0190 | 2755±71.7 | 1633±81.9 | 0.008±0.0011 | 22.7±0.73 | 2.19±0.153 |

Table S14 Micronutrient concentrations in grass of the third cut

| Treatments | Cd (mg kg^-1^) | Cu (mg kg^-1^) | Fe (mg kg^-1^) | Mn (mg kg^-1^) | Mo (mg kg^-1^) | P (mg kg^-1^) | S (mg kg^-1^) | Se (mg kg^-1^) | Zn (mg kg^-1^) | N (% DM) |
| --- | --- | --- | --- | --- | --- | --- | --- | --- | --- | --- |
| A-CK | 0.024±0.0023 | 5.43±0.158 | 90.7±32.00 | 166±11.4 | 0.878±0.0877 | 3486±126.9 | 2660±80.8 | 0.064±0.0044 | 17.6±0.50 | 1.39±0.017 |
| A-U-O | 0.046±0.0038 | 6.02±0.235 | 54.2±3.11 | 127±11.1 | 0.334±0.0207 | 3374±155.9 | 1607±95.0 | 0.037±0.0018 | 18.7±0.33 | 1.39±0.060 |
| A-U-I | 0.056±0.0031 | 6.12±0.446 | 56.3±9.40 | 132±15.7 | 0.407±0.0704 | 3350±290.5 | 1476±84.9 | 0.035±0.0028 | 19.2±0.81 | 1.41±0.030 |
| A-F-O | 0.021±0.0028 | 5.23±0.169 | 76.8±21.41 | 125±13.9 | 1.556±0.1876 | 3730±118.5 | 2644±168.8 | 0.061±0.0179 | 16.7±0.41 | 1.44±0.028 |
| A-F-I | 0.021±0.0007 | 5.47±0.145 | 69.7±5.64 | 134±14.1 | 1.475±0.0751 | 3829±93.3 | 2735±98.3 | 0.047±0.0026 | 17.7±0.41 | 1.50±0.067 |
| A-UF-O | 0.036±0.0021 | 6.79±0.129 | 56.1±3.55 | 116±9.8 | 1.241±0.0358 | 4396±78.5 | 1816±44.4 | 0.046±0.0075 | 19.9±0.29 | 1.50±0.024 |
| A-UF-I | 0.049±0.0057 | 6.95±0.348 | 61.0±5.57 | 126±3.3 | 1.148±0.0989 | 4154±128.3 | 1666±42.0 | 0.053±0.0112 | 21.4±1.43 | 1.46±0.027 |
| G-CK | 0.047±0.0011 | 5.04±0.206 | 145±67.4 | 149±4.6 | 1.137±0.0682 | 2987±139.0 | 1366±73.9 | 0.019±0.0046 | 19.7±0.71 | 1.22±0.020 |
| G-U-O | 0.067±0.0097 | 5.73±0.350 | 67.7±13.98 | 105±12.8 | 0.519±0.1073 | 3306±137.1 | 1623±95.6 | 0.026±0.0108 | 21.1±1.62 | 1.40±0.033 |
| G-U-I | 0.060±0.0052 | 5.37±0.180 | 69.5±13.72 | 93.3±8.74 | 0.369±0.0747 | 3030±98.0 | 1534±37.2 | 0.013±0.0014 | 20.0±1.23 | 1.30±0.017 |
| G-F-O | 0.047±0.0031 | 5.07±0.137 | 86.8±23.20 | 95.7±6.89 | 1.974±0.0656 | 3266±81.2 | 1827±61.3 | 0.014±0.0013 | 17.9±0.32 | 1.28±0.042 |
| G-F-I | 0.044±0.0036 | 4.85±0.085 | 146±53.3 | 105±3.9 | 1.779±0.1615 | 3169±61.5 | 1837±146.5 | 0.017±0.0058 | 16.3±0.76 | 1.23±0.036 |
| G-UF-O | 0.058±0.0051 | 5.76±0.175 | 71.6±1.25 | 72.9±7.10 | 0.983±0.1245 | 3271±81.0 | 1549±35.2 | 0.023±0.0108 | 21.0±0.64 | 1.39±0.045 |
| G-UF-I | 0.055±0.0057 | 5.21±0.309 | 56.8±3.36 | 72.6±7.96 | 0.801±0.1281 | 3173±112.5 | 1470±61.6 | 0.011±0.0011 | 19.9±1.03 | 1.35±0.036 |

**References**

Doak B (1952) Some chemical changes in the nitrogenous constituents of urine when voided on pasture. J Agric Sci 42: 162-171. <https://doi.org/10.1017/S0021859600058767>

Martens DA, Suarez DL (1996) Selenium speciation of soil/sediment determined with sequential extractions and hydride generation atomic absorption spectrophotometry. Environ Sci Technol 31: 133-139. <https://doi.org/10.1021/es960214>

Martens DA, Suarez DL (1997) Selenium speciation of marine shales, alluvial soils, and evaporation basin soils of California. J Environ Qual 26: 424-432. <https://doi.org/10.2134/jeq1997.00472425002600020013x>

Sears PD, Goodall VC, Newbold RP (1942) The effect of sheep droppings on yield, botanical composition, and chemical composition of pasture. 1. Establishment of trial, technique of measurement, and results for the 1940-41 season. New Zealand Journal of Science and Technology Section A: 36-61.

Stroud JL, McGrath SP, Zhao FJ (2012) Selenium speciation in soil extracts using LC-ICP-MS. Int J Environ Anal Chem 92: 222-236. <https://doi.org/10.1080/03067310903111661>

White E (1960) The distribution and subsequent disappearance of sheep dung on Pennine moorland. J Anim Ecol: 243-250. <https://doi.org/10.2307/2202>

Williams PH, Haynes RJ (1990) Influence of improved pastures and grazing animals on nutrient cycling within New Zealand soils. N Z J Ecol 14: 49-57. <https://www.jstor.org/stable/24053311>
